# NK Cell‐Derived Small Extracellular Vesicles Armed With CLDN4‐Targeting Peptides Potentiate Radiotherapy in Gastric Cancer

**DOI:** 10.1002/jev2.70200

**Published:** 2025-11-18

**Authors:** Anqi Dong, Wenhao Shen, Xiaochun Shen, Shu Liu, Dongbao Li, Min Li, Minghui Li, Yan Ma, Jin Zhou, Lin Hu, Kai Yang

**Affiliations:** ^1^ Department of Pathology, the First Affiliated Hospital, State Key Laboratory of Radiation Medicine and Protection, School of Radiation Medicine and Protection & School for Radiological and Interdisciplinary Sciences (RAD‐X), Collaborative Innovation Center of Radiation Medicine of Jiangsu Higher Education Institutions, Cancer Institute, Suzhou Medical College Soochow University Suzhou Jiangsu China; ^2^ Department of Central Laboratory and Oncology Taizhou People's Hospital Affiliated to Nanjing Medical University Taizhou Jiangsu China; ^3^ Department of Respiratory, the First Affiliated Hospital of Soochow University Soochow University Suzhou Jiangsu China; ^4^ Department of General Surgery, the First Affiliated Hospital of Soochow University Soochow University Suzhou Jiangsu China; ^5^ Institutes of Biology and Medical Sciences Soochow University Suzhou Jiangsu China; ^6^ MOE Key Laboratory of Geriatric Diseases and Immunology, School of Biology and Basic Medical Sciences Soochow University Suzhou Jiangsu China; ^7^ Department of Pathology, the First Affiliated Hospital of Soochow University Soochow University Suzhou Jiangsu China

**Keywords:** CLDN4, gastric cancer organoids, natural killer cell‐derived small extracellular vesicles, radiotherapy, tumour‐targeting

## Abstract

Gastric cancer (GC) persists as one of the most lethal malignancies globally, primarily due to late‐stage diagnosis, limited therapeutic targeting options, and inherent resistance to conventional therapies. While molecular profiling has advanced our understanding of GC, the development of effective delivery systems capable of precise tumour targeting and enhanced treatment response remains an unmet need. In this work, we explored targeted therapeutic approaches for GC by leveraging patient‐derived organoid models. Firstly, we confirmed claudin‐4 (CLDN4) as an overexpressed target in malignant epithelial cells in GC through comprehensive analysis of multiple single‐cell RNA sequencing datasets. Capitalising on this discovery, we developed an innovative nano‐therapeutic biomaterial, designated NESC (NK‐sEV‐SpoVM‐c‐CPE^Q317I^), by engineering natural killer cell‐derived small extracellular vesicles (NK‐sEVs) with a CLDN4‐targeting peptide and a membrane‐curvature‐sensing domain. Multimodal imaging further confirmed tumour‐specific accumulation of NESC, underscoring its targeting precision. Proteomic profiling and functional assays revealed that NK‐sEVs possessed intrinsic radiosensitising properties, which were significantly augmented upon conjugation with the targeting peptide. The resulting NESC platform demonstrated robust tumour‐suppressive activity and enhanced radiosensitisation in both GC organoids and organoid‐derived xenograft models. Collectively, by harnessing patient‐derived organoids for functional validation, this study not only establishes a versatile framework for developing targeted sEV‐based therapeutics but also provides a translational foundation for future clinical applications in GC management.

## 1 Introduction

Gastric cancer (GC) ranks as the fifth most prevalent malignancy and the third leading cause of cancer‐related mortality worldwide (Sung et al. 2021). Despite advances in therapeutic strategies—including surgical resection, chemotherapy, molecularly targeted therapies, and immunotherapy—the global 5‐year survival rate for GC remains disappointingly low (Machlowska et al. 2020; Joshi and Badgwell 2021). This underscores the urgent need for novel research approaches and innovative treatment modalities. In this context, three‐dimensional organoid models have emerged as powerful tools for disease modelling, drug discovery, and personalised medicine. They have emerged as versatile tools for disease modelling, drug discovery and personalised medicine (Zhao et al. 2022). These self‐organising in vitro systems faithfully recapitulate the architectural and functional complexity of their tissue of origin. In this study, GC organoids were utilised to develop a targeted delivery strategy based on a bioengineered extracellular vesicle material and further potentiated its efficacy by combining it with radiotherapy.

Recent investigations have highlighted the claudin (CLDN) protein family, comprising at least 27 members, as promising therapeutic targets in GC (Tsukita et al. 2019). The abnormal expression of CLDNs can lead to the dysfunction of epithelial barrier and loss of epithelial polarity, which can result in a variety of cancers, including GC (Hashimoto and Oshima 2022). Among the CLDN family, CLDN18.2 has attracted particular attention, with interim clinical trial data demonstrating therapeutic benefits from CLDN18.2‐targeted approaches, including the monoclonal antibody zolbetuximab and chimeric antigen receptor (CAR) T‐cell therapy (Qi et al. 2022; Alsina et al. 2023; Shitara et al. 2023). Additionally, radiolabeled anti‐CLDN18.2 antibodies have shown promise for both diagnostic imaging and radio‐immunotherapy in GC patients and xenograft models (Wang et al. 2023; Qi et al. 2024; Zeng et al. 2023). Parallel developments have identified CLDN6 as another viable target, with clinical trials reporting favourable responses to CLDN6‐directed CAR T‐cell therapy in solid tumours, including GC (Mackensen et al. 2023), and preclinical studies demonstrating tumour regression with CLDN6‐targeted antibody‐drug conjugates (McDermott et al. 2023). Cocoo et al. carried out extensive research on a novel peptide derived from the carboxy‐terminal fragment of Clostridium perfringens enterotoxin (c‐CPE), which could target CLDN3 and CLDN4, and contribute to in vivo visualisation of and suicide gene delivery to ovarian cancer (Cocco et al. 2015; Cocco et al. 2017). These collective findings position the CLDN family as a compelling target class for GC therapeutics.

**SCHEME 1:**
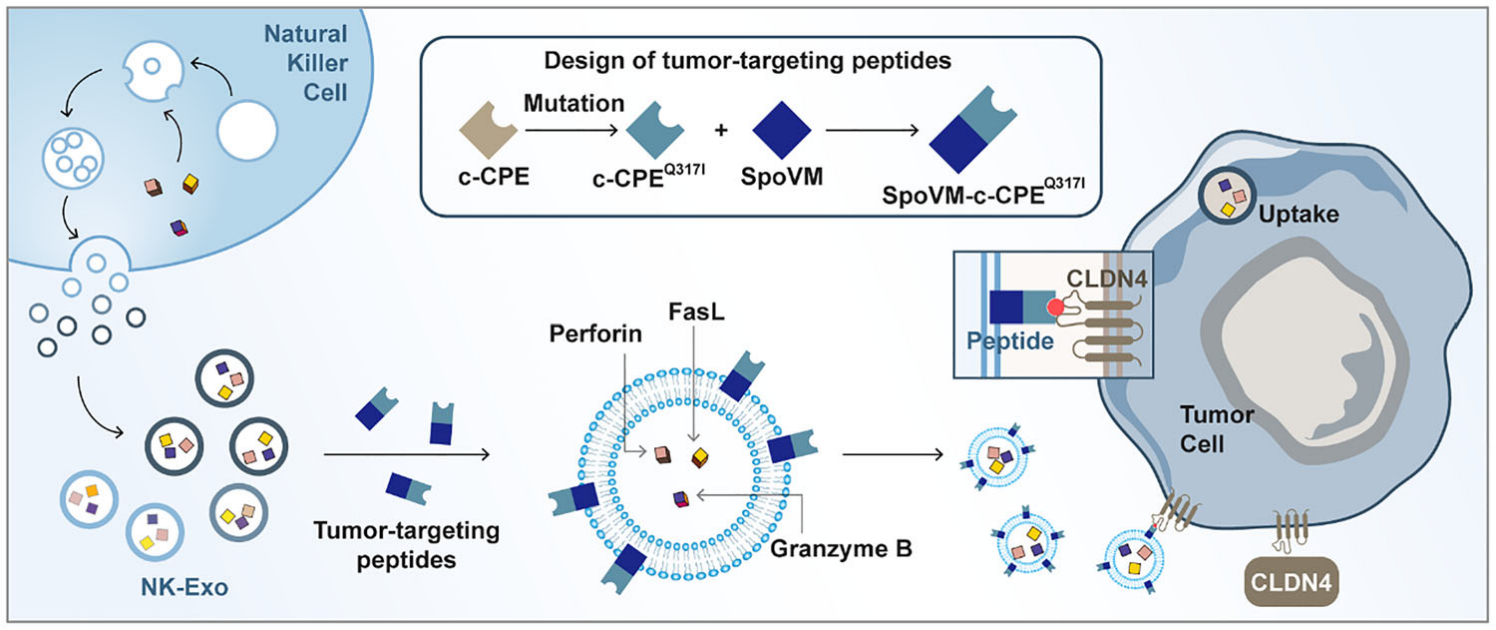
Schematic diagram of the design of NK‐sEV‐SpoVM‐c‐CPE^Q317I^ (NESC) and its targeted delivery to GC.

Natural killer (NK) cells are innate immune lymphoid cells that play a vital role in the anti‐cancer immune response (Shimasaki et al. 2016). Their cytotoxic mechanisms involve two ways: one involves membrane‐disrupting proteins, including perforin and granzyme B, and the other involves cell death ligands, namely the Fas ligand (FasL) and tumour necrosis factor (TNF)‐related apoptosis‐inducing ligand (TRAIL) (Wu et al. 2020; Alizadeh Zeinabad et al. 2023; Li et al. 2023). Moreover, NK cell‐derived small extracellular vesicles (NK‐sEV), 30–200 nm lipid bilayer vesicles that transfer bioactive cargo between cells, have recently emerged as attractive delivery vehicles for cancer therapy (Liang et al. 2021; Kim et al. 2022; Zhu et al. 2017; Liang et al. 2020; Wang et al. 2021; Wan et al. 2018). In this work, therefore, on the basis of our findings of high CLDN4 expression on GC by single‐cell transcriptomics, we designed a novel biomaterial based on NK‐sEVs for targeting GC overexpressing CLDN4 to enhance the therapeutic efficiency of GC (Scheme 1). In this treatment strategy, we designed a high‐affinity peptide c‐CPE^Q317I^ by computational mutation prediction, which was then conjugated to the membrane curvature‐sensing SpoVM peptide (Kim et al. 2017; Peluso et al. 2019; Fu et al. 2015), thereby stably anchoring to NK‐sEVs to form NK‐sEV‐SpoVM‐c‐CPE^Q317I^ (NESC). Subsequently, we have demonstrated that this biomaterial could target the GC cells and induce significant cytotoxic effects on GC cell lines, patient‐derived GC organoids, and organoid‐based tumours in mice. More importantly, we found that NESC could significantly upregulate the intracellular reactive oxygen species (ROS) levels and further enhance the therapeutic efficiency of radiotherapy. Therefore, our work for the first time demonstrated that targeted NESC combined with radiotherapy provided a new approach for the clinical precision treatment of GC.

## 2 Methods and Materials

### 2.1 In Silico Analysis

The GC scRNA‐seq datasets were downloaded from the Gene Expression Omnibus (GEO) database via the accession numbers GSE183904, GSE134520, GSE163558 and GSE167297 or acquired from a previous study by Sathe et al. (Sathe et al. 2020). With the Python module Scanpy (Wolf et al. 2018), genes expressed by fewer than three cells and cells expressing fewer than 200 genes were excluded for the next steps. Cells with more than 20% mitochondrial RNA were also excluded. Counts per million (CPM) normalisation was carried out with a target sum of 10,000, and then the data matrix was log transformed. After regressing out unwanted sources of variation and scaling to unit variance and zero mean, principal component analysis (PCA) was performed and then visualised using the uniform manifold approximation and projection (UMAP) method. The Leiden algorithm was used to cluster cells into subgroups with a resolution of 0.1. The Python module infercnvpy (https://github.com/icbi‐lab/infercnvpy) was used to distinguish malignant from benign cells by calculating the copy number variation (CNV) of chromosomes. Cells with a CNV score higher than average were defined as malignant cells, while the others were supposed to be non‐malignant ones. Differentially expressed genes (DEGs) between benign and malignant cells were identified by the rank‐gene‐groups function in Scanpy. DEGs of normal and tumour tissues in The Cancer Genome Atlas Program (TCGA) were analysed using GEPIA (Tang et al. 2017).

### 2.2 Immunohistochemistry (IHC)

Clinical specimens of paired normal and tumour tissue were embedded in paraffin and then cut into 5 µm‐thick sections. IHC staining was performed according to the instructions of the CLDN4‐specific rabbit monoclonal antibody (Cat# ab210796) purchased from Abcam (USA). The IHC sections were scanned and analysed with NanoZoomer S60 (Hamamatsu Photonics) and ImageJ. The product of the positively stained area in the total area and the positive degree was taken as the final score.

### 2.3 Model Construction and Mutation Prediction

The crystal structure of human CLDN4 in complex with the *Clostridium perfringens* enterotoxin C‐terminal domain (PDB ID: 7KP4) was used for the calculation. A CLDN4‐peptide complex was constructed using the amino acid sequence from residue 290 to 319 from the c‐CPE chain. The effects of all possible mutations in this peptide sequence on the CLDN4‐CPE complex and the CLDN4‐c‐CPE complex were then calculated. Two widely recognised prediction methods, FoldX (Guerois et al. 2002) and BeAtMuSiC (Dehouck et al. 2013), were utilised for these calculations.

### 2.4 Cell Lines and Cell Culture

Human NK cell line NK‐92MI, human GC cell lines AGS, MGC803, MKN45, NCI‐N87, SGC7901, SNU‐1 and normal human gastric epithelial cell line GES‐1 were purchased from Procell Life Science & Technology Co., Ltd. (Wuhan, China). NK‐92MI were cultured in MEMα, containing 0.2 mM inositol, 0.1 mM β‐mercaptoethanol, 0.02 mM folic acid, 12.5% horse serum, 12.5% fetal bovine serum (FBS) and 1% penicillin/streptomycin (P/S). The other cell lines were cultured in RPMI‐1640 supplemented with 10% FBS and 1% P/S. All cell lines were kept under a humidified atmosphere containing 5% CO_2_ and 95% O_2_ at 37°C or stored in serum‐free cell freezing medium in liquid nitrogen.

### 2.5 EV Isolation and Characterisation

NK‐sEVs were isolated by sequential centrifugation of NK‐92MI culture medium at 300 × *g* for 10 min, 2000 × *g* for 10 min at 4°C to remove residual cells and dead cells, respectively. The supernatants were then filtered through 0.22 µm syringe filters to remove cell debris. The filtrate was ultra‐centrifuged at 100,000 × *g* for 70 min at 4°C and re‐suspended in phosphate‐buffered saline (PBS). Another ultra‐centrifugation under the same conditions was conducted, resulting in purified NK‐sEV.

Transmission electron microscopy (TEM) was performed to verify the presence of purified NK‐sEVs. A 10 µL sample of NK‐sEVs was placed on Formvar carbon‐coated copper grids (400 mesh) and dried at room temperature for 4 h. Afterwards, the sample was negatively stained with 10 µL of phosphotungstic acid solution (2%, pH 7.0) three times. After each staining, 4–6 h of drying at room temperature was required. The morphology of NK‐sEVs was observed using a FEI Tecnai G2 F20 field emission transmission electron microscope operated at 80 kV. Nanoparticle tracking analysis (NTA) was performed to analyse the particle size distribution of purified NK‐sEVs by Zetasizer Nano ZS90 (Malvern Panalytical Ltd., UK). The purified sEVs from NK‐92MI were suspended in PBS to a concentration of 50 µg protein/mL. 80 µL was used in the cuvette for the analysis. 10 replicate measurements were made for each batch.

### 2.6 EV Labelling and Internalisation

NK‐sEVs were labelled with the fluorescent dye PKH67 (Sigma‐Aldrich, USA) according to the manufacturer's instructions. In detail, 10 µL of condensed NK‐sEVs and 0.5 µL of PKH67 dye were dissolved in 125 µL Diluent C, respectively and then mixed together. After 5 min of incubation, excess PKH67 was removed by ultrafiltration using an Amicon Centrifugal Filtration Unit with a molecular weight cutoff of 100 kDa at 4000 × *g* for 15 min.

To study the cellular uptake of NK‐sEVs, PKH67‐labelled NK‐sEVs were diluted in PBS and then incubated with GC cells for 6 h. Afterwards, the medium was removed and the cells were washed with PBS and fixed with 4% paraformaldehyde. All slides were mounted with fluorescence‐protecting mounting medium containing nuclei dye DAPI (Beyotime Biotechnology, China). Cell images were captured by an Olympus confocal microscopy system (FV1200) and then further analysed by ImageJ software (Ver. 1.54d, NIH, Bethesda, MD, USA).

### 2.7 Western Blot

Cell lysates were lysed with radioimmunoprecipitation assay (RIPA) buffer containing 1% protease inhibitor for 30 min on ice. The protein lysates were separated by 10% sodium dodecyl sulfate–polyacrylamide gel electrophoresis (SDS‐PAGE), transferred onto polyvinylidene difluoride membranes (PVDF; Millipore, MA, USA), and blocked with 5% skimmed milk (Sigma‐Aldrich, USA) in Tris‐buffered saline with 0.1% Tween‐20 (TBST; pH 7.4) at room temperature for 1 h. The membranes were then subsequently incubated with specific primary antibodies at 4°C overnight and horseradish peroxidase (HRP)‐conjugated secondary antibodies at room temperature for 1 h. The blots were pictured using a Tanon 5200CE Chemi‐Image System (Abclonal, Wuhan, China) and quantified using ImageJ software.

The following antibodies were used. CD63 antibody (Cat# 48772), TSG101 antibody (Cat# 49270) and Calnexin antibody (Cat# 49102) were purchased from Signalway Antibody LLC (Maryland, USA). CLDN4 antibody (Cat# ab210796) was obtained from Abcam (USA). PRF1 (Cat# 222602), GZMB (Cat# 252579) and FasL (Cat# 370112) antibodies were purchased from ZenBio (Chengdu, China). β‐actin antibody (Cat# GB11001), HRP‐conjugated goat‐anti‐rabbit (Cat# GB23303) or mouse (Cat# GB23301) antibodies were purchased from Servicebio (Wuhan, China).

### 2.8 Liquid Chromatography–Mass Spectrometry (LC‐MS) and Proteomic Bioinformatic Analysis

NK‐sEVs were independently isolated in three parallel preparations and subjected to LC‐MS analysis. Proteins of NK‐sEVs were separated by SDS‐PAGE. Protein bands were excised from Coomassie‐stained SDS‐PAGE gels using a clean scalpel. The excised gel pieces were further cut into approximately 1 × 1 mm fragments to facilitate efficient reagent penetration, and were transferred into low‐protein‐binding microcentrifuge tubes for subsequent in‐gel digestion. Only peptides/proteins that were consistently identified in at least two of the three replicates were considered as valid identifications. The sEV‐depleted culture medium supernatant of NK‐92MI cells was used as a control group. Only proteins uniquely present in the NK‐sEV samples after subtraction of control‐derived proteins were retained to define the final EV‐specific proteome. The raw data were processed using the MaxQuant search engine (http://maxquant.org/) and searched within the human proteome database (https://www.uniprot.org/proteomes/UP000005640). Kyoto Encyclopedia of Gene and Genomes (KEGG) pathway enrichment analysis of DEGs was performed to uncover the potential biological function using the Metascape database (http://metascape.org/).

### 2.9 Cell Viability

To evaluate the cell viability of NCI‐N87 cells in the presence of NK‐sEV, a cell counting kit‐8 (CCK‐8) assay (Beyotime Biotechnology, Shanghai, China) was performed according to the manufacturer's instructions. Briefly, cells were seeded in 96‐well plates (4 × 10^4^ cells/well) and co‐cultured with NK‐sEVs to evaluate in a dose‐dependent manner. Then, 10 µL of the CCK‐8 solution was added to each well and the plates were incubated at 37 °C for 4 h. After incubation, the absorbance was measured at 450 nm using a microplate reader (Agilent, USA). The viability rate of the co‐cultured cells was calculated relative to that of control cells.

### 2.10 GC Organoids (GCOs)

GC tissue was collected from voluntary GC patients who had undergone abdominal surgery. All the reagents needed for storage, cultivation and digestion of organoids were purchased from bioGenous Technologies (Suzhou, China), and experiments concerning organoids were conducted under the guidance of its technical manual. NESC was directly added to the culture medium of the GCOs, followed by incubation under standard culture conditions.

### 2.11 In Vivo Experiments

To establish mouse models bearing subcutaneous tumours, NCI‐N87 (1 × 10^6^) cells were suspended in 50 µL PBS and injected into BALB/c nude mice (6–8 weeks) subcutaneously. For in vivo imaging, tumour‐bearing mice were i.v. injected with Cy5.5‐labelled NESC (100 µg). Whole‐body fluorescence imaging was performed at 1, 4, 12, 24 and 48 h after the injection using the in vivo imaging system (IVIS, PerkinElmer, USA). Mice were anaesthetised with isoflurane during imaging. Forty‐eight hours after the injection, the mice were sacrificed, and major organs, including lungs, heart, liver, spleen and kidneys, were collected for biodistribution analysis by ex vivo fluorescence imaging. For SPECT/CT imaging, tumour‐bearing mice received ^125^I‐labeled NESC (100 µg, 200 µCi per mouse) via tail vein injection and were scanned by a microSPECT/CT system (MILabs, Utrecht, the Netherlands) under isoflurane anaesthesia. To evaluate the antitumour effect of NESC, tumour‐bearing mice were randomised into four treatment groups: (1) control (PBS), (2) NESC alone, (3) x‐ray alone, and (4) NESC plus x‐ray combination therapy. The tumour volume and the weight of the mice were monitored every 2 days. Mice were sacrificed when the tumour volume exceeded 1000 mm^3^. All of the mouse experiments were performed according to the protocols approved by Soochow University Laboratory Animal Centre.

### 2.12 Statistical Analysis

Experiments were performed in a randomised and double‐blinded manner. All continuous data are based on the mean ± standard deviation (mean ± SD). The statistical software GraphPad Prism 9 was used to compare the differences in each group. Student's *t*‐test was used for analysing the differences between the experimental groups. *p* < 0.05 was considered statistically significant.

## 3 Results

### 3.1 In Silico and Ex Vivo Analyses of CLDN4 Expression in GC

**FIGURE 1:**
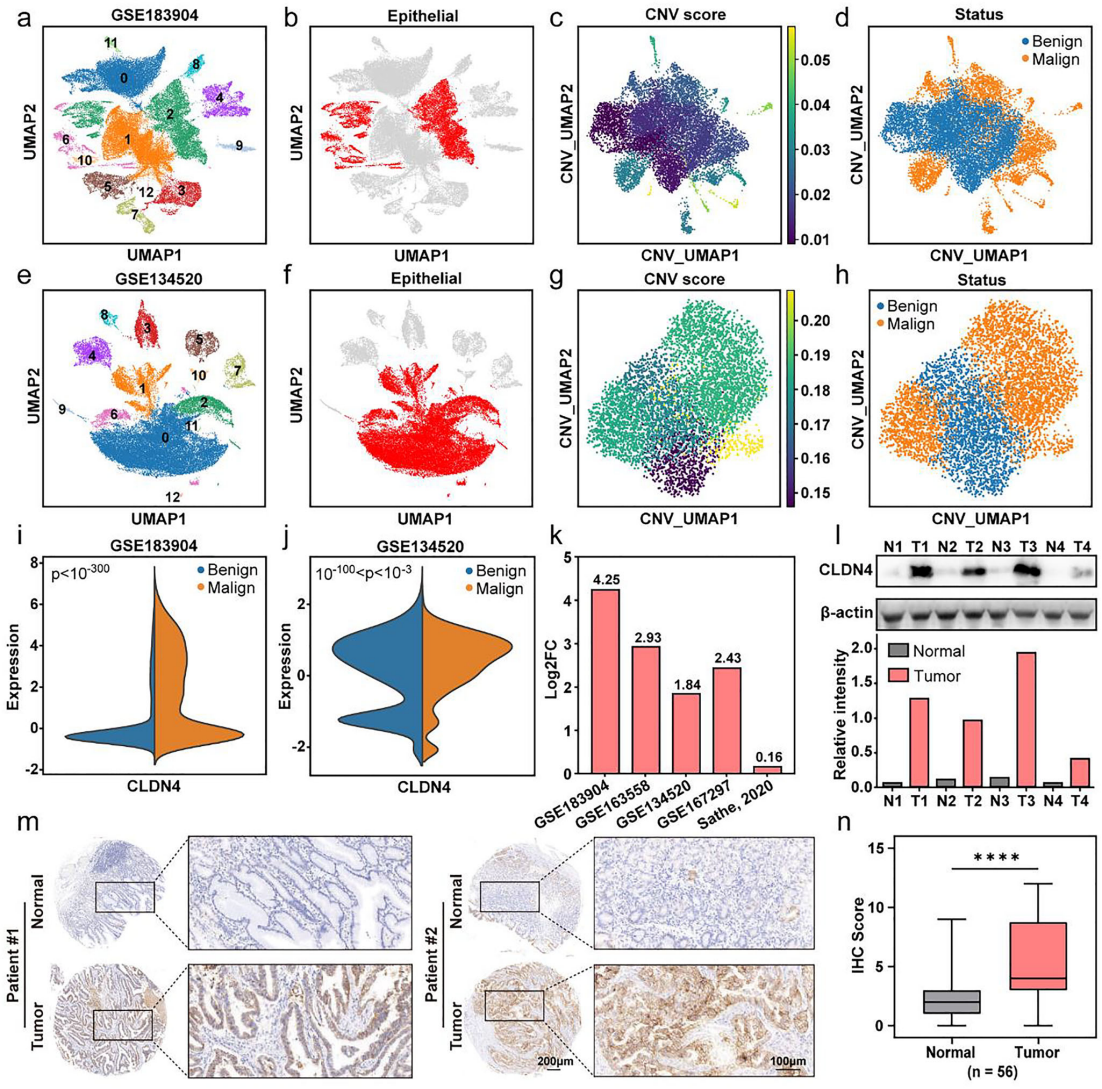
In silico and ex vivo analyses of CLDN4 in GC. (a and e) Uniform manifold approximation and projection (UMAP) plot of 50,061 cells of GSE183904 (a) and GSE134520 (e) colored by cluster identity. (b and f) UMAP plot of GSE183904 (b) and GSE134520 (f), highlighting epithelial cells (red) versus non‐epithelial cells (grey). (c and g) UMAP plot depicting the CNV scores of all epithelial cells in GSE183904 (c) and GSE134520 (g). (d and h) UMAP plot showing the classification of malignant and non‐malignant epithelial cells in GSE183904 (d) and GSE134520 (h). (I and j) Half violin plot showing CLDN4 expression in putative malignant and non‐malignant cells in GSE183904 (i) and GSE134520 (j). (k) Log2 fold‐change of CLDN4 expression in malignant epithelial cells versus non‐malignant epithelial cells in all five datasets. (l) Western blot of CLDN4 in 4 pairs of GC patients’ samples. (m) Representative pictures of IHC staining of CLDN4 in GC patients. (n) Boxplot showing scores of IHC staining of CLDN4 in GC patients (*n* = 56). *p* values were calculated by paired *t*‐test (****p < 0.0001).

Single‐cell RNA sequencing (scRNA‐seq) datasets comprising paired normal and tumour tissues from gastric cancer (GC) patients were acquired from the Gene Expression Omnibus (GEO) database and a previous study by Sathe et al. (Sathe et al. 2020). Following rigorous quality control, the GSE183904 dataset retained 50,061 cells and 23,554 genes, while the GSE134520 dataset contained 36,619 cells and 22,910 genes. After normalisation, scaling, and principal component analysis (PCA), we identified 13 distinct cell clusters in GSE183904 and 12 clusters in GSE134520 (Figure 1a,e). Using established marker genes for epithelial (EPCAM, KRT18, KRT19, CDH1, MUC1) and non‐epithelial (VIM, ACTA2, CD4, PTPRC) cells (Figure S1a,b), we successfully segregated these cell populations and subsequently focused our analysis on epithelial cells (Figure 1b, f). We then calculated copy number variation (CNV) scores for all epithelial cells across five datasets (Figure 1c,g), establishing dataset‐specific thresholds based on mean CNV scores (GSE183904: 0.0177695; GSE134520: 0.0178430; GSE163558: 0.0214949; GSE167297: 0.0093554; Sathe et al.: 0.0081790) to distinguish malignant from non‐malignant cells (Figure 1d,h). Differentially expressed genes (DEGs) analysis, ranked by *t*‐values, consistently identified CLDN4 as the top upregulated gene in malignant epithelial cells compared to their non‐malignant counterparts in the GSE183904 dataset (Table S1). This pattern was corroborated in the GSE134520 dataset (Figure 1i,j) and replicated across three additional datasets, with log2 fold‐change values ranging from 0.16 to 4.25 (Figures S2–S4), suggesting CLDN4's potential as a discriminative marker for malignant cell populations in GC (Figure 1k).

To validate these findings, we examined four paired tumour and adjacent normal tissue samples from GC patients who underwent gastrectomy. Western blot analysis demonstrated significantly elevated CLDN4 expression in tumour tissues (Figure 1l). Furthermore, a tissue microarray containing 56 cases was used for immunohistochemistry (IHC) staining to determine CLDN4 expression pattern in paired GC tissues and adjacent normal tissues (Table S2). Consistent with the previous analyses, CLDN4 was highly expressed in GC tissues (Figure 1m), and IHC scores were also significantly higher (Figure 1n). Meanwhile, various kinds of normal and tumour tissues from TCGA were also examined. Stomach adenocarcinoma (STAD) data showed that GC tissues expressed higher levels of CLDN4 than normal tissues (Figure S5a). Across all cancer types, CLDN4 expression was generally elevated in tumour tissues relative to normal counterparts, with the exception of head and neck squamous cell carcinoma (HNSC), kidney renal clear cell carcinoma (KIRC), sarcoma (SARC) and skin cutaneous melanoma (SKCM) (Figure S5b). These findings suggest that CLDN4 may serve as a promising candidate for theranostic applications in GC.

### 3.2 Design of CLDN4‐Targeting NK‐sEV‐Peptide Complexes

**FIGURE 2:**
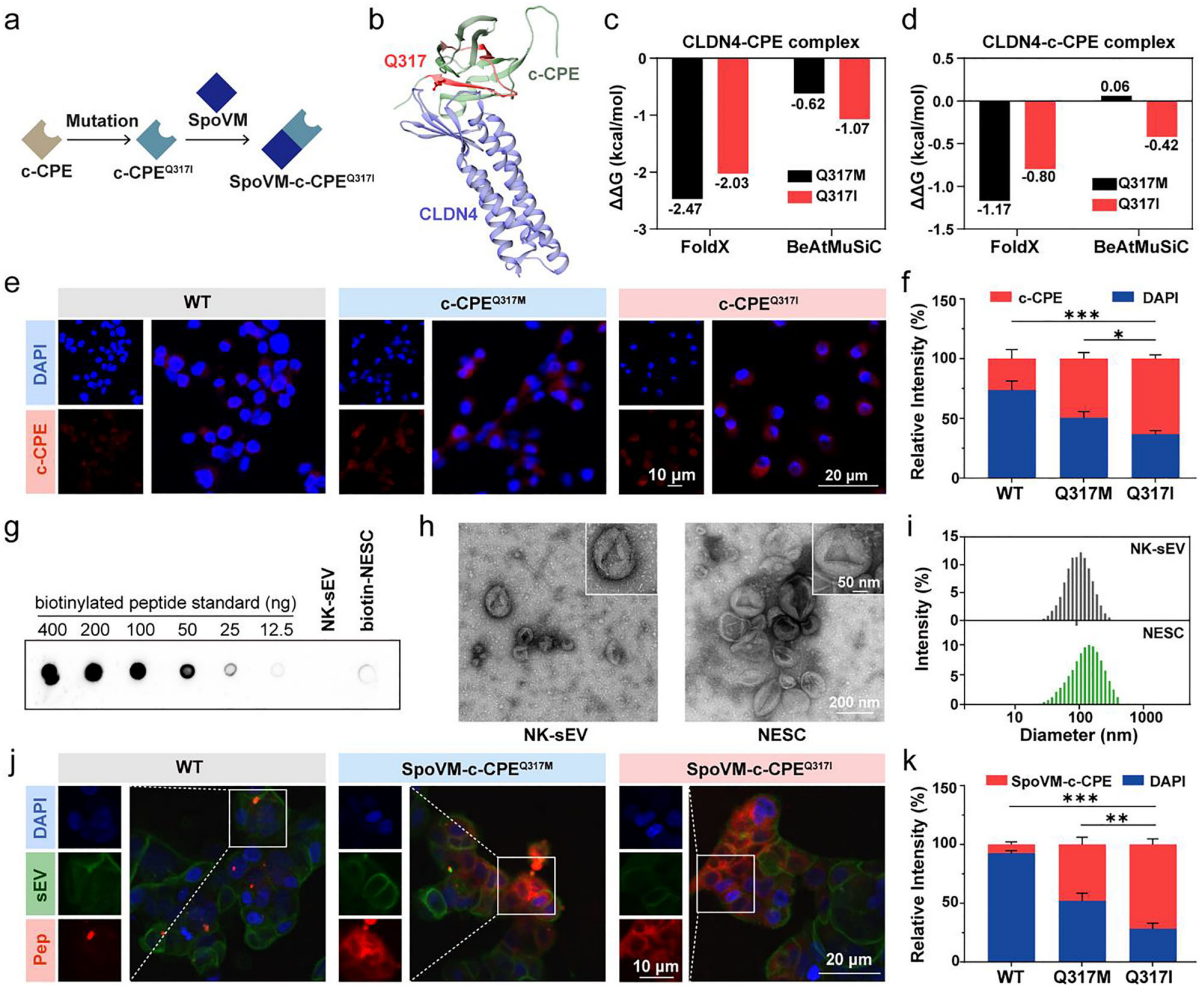
Design of tumour‐targeting peptide and isolation of NK‐sEV. (a) Illustration of the sequence structure of the SpoVM‐c‐CPE^Q317I^ peptide. (b) Diagram of CLDN4‐c‐CPE complex illustrating the location of the mutated residue (Q317I). (c and d) The binding affinity changes of CLDN4‐CPE complex (c) and CLDN4‐c‐CPE complex (d) predicted by FoldX and BeAtMuSiC. (e and f) Representative confocal images of wildtype c‐CPE and its mutants, c‐CPE^Q317M^ and c‐CPE^Q317I^, binding to NCI‐N87 cells (e) (blue: DAPI, red: peptides) and relative fluorescence intensities measured by ImageJ software (f). (g) Dot blot analysis of NK‐sEVs ligated with biotinylated peptide. Peptides with known concentration were used as a reference for quantification. A particle analyser was used to obtain the number of EVs loaded per dot. (h) TEM images of NK‐sEVs and NESC. (i) Size distribution of NK‐sEVs and NESC measured by NTA. (j and k) Representative confocal images of SpoVM‐c‐CPE and its mutants, SpoVM‐c‐CPE^Q317M^ and SpoVM‐c‐CPE^Q317I^, anchoring to NK‐sEVs and then binding to NCI‐N87 cells (j) (blue: DAPI, green: NK‐sEV, red: peptides). Relative fluorescence intensities were measured by ImageJ software (k). *p* values were calculated by unpaired *t*‐test (*p < 0.05, **p < 0.01, ***p < 0.001).

In this study, we initially designed a linear peptide by fusing the sequences of SpoVM and c‐CPE, resulting in the formation of the SpoVM‐c‐CPE peptide (Figure 2a). To further optimise the binding affinity of SpoVM‐c‐CPE towards CLDN4, we performed site‐directed mutagenesis on c‐CPE and computationally evaluated the binding free energy changes (ΔΔG) between wild‐type and mutant peptides. Two independent algorithms, FoldX and BeAtMuSiC, were employed to predict the structural and energetic effects of point mutations on the CLDN4‐c‐CPE interaction (Figure 2b). Mutants with ΔΔG < 0 kcal/mol were prioritised as high‐affinity candidates. Among all 570 possible mutants in the CPE sequences, FoldX identified Q317M as the most stabilising mutation (lowest ΔΔG), whereas BeAtMuSiC predicted Q317I as optimal. However, the ΔΔG values for both mutations were within the range of highly stabilising mutations predicted by two methods (Figure 2c). Additionally, the ΔΔG values of the CLDN4‐c‐CPE peptide complex were also calculated by these two methods. The results indicated that the ΔΔG value was negative in at least one of the two methods (Figure 2d). Importantly, mutation site Q317 of c‐CPE was positioned at the protein‐protein interface, suggesting that mutations at this site could potentially modulate the binding affinity between the two molecules. Therefore, we selected two mutants, c‐CPE^Q317I^ and c‐CPE^Q317M^, for further experimental validation.

After determining the key mutation site of c‐CPE, we next investigated the binding ability of the two c‐CPE mutants to GC cells. Among all the GC cell lines examined, NCI‐N87 exhibited the highest level of CLDN4 expression and was therefore selected for subsequent experiments (Figure S6a). As shown in Figure 2e,f, both c‐CPE^Q317I^ and c‐CPE^Q317M^ exhibited enhanced cell‐binding abilities compared with the wild‐type c‐CPE, with c‐CPE^Q317I^ demonstrating superior performance over c‐CPE^Q317M^. These findings were consistent with the prior computational predictions. Based on the optimised c‐CPE variant, we subsequently conjugated it to the N‐terminus of the SpoVM sequence, thereby generating the SpoVM‐c‐CPE^Q317I^ peptide, which was sequenced as: MKFYTIKLPKFLGGIVRAMLGSFRKDSLDAGQYVLVMKANSSYSGNYPYSILFIKF.

Next, we prepared the NK‐sEVs by a series of sequential steps including several centrifugations and filtration procedures (Figure S6a). The morphology of NK‐sEVs was examined by transmission electron microscope (TEM), showing uniform spheres with a diameter of 50–150 nm (Figure 2h, left panel). Successful NK‐sEV isolation was further demonstrated by the expression of CD63, TSG101 and HSC70(Figure S6c). Calnexin was used as a negative control. NK‐sEV accumulation on recipient cells was then proved by confocal microscopy (Figure S6d). After successfully obtaining NK‐sEV, the mutated SpoVM‐c‐CPE^Q317I^ peptide could be adsorbed and embedded in the membranes of NK‐sEVs to generate NK‐sEV‐SpoVM‐c‐CPE^Q317I^ (NESC), which could then be specifically enriched on the membrane surface of GC cells with high expression of CLDN4. To quantify the number of peptides that were conjugated to NK‐sEVs, we designed biotinylated peptides and conjugated them to NK‐sEVs. Afterwards, we compared the biotin signals from the biotin‐NESC to a serial dilution of peptides with known concentration. This comparison indicated that there were ∼210 copies of peptides ligated to each NK‐sEV on average (Figure 2g). The TEM image showed no significant changes in appearance compared with NK‐sEVs without peptides (Figure 2h, right panel). NK‐sEV size distribution measured by nanoparticle tracking analysis (NTA) revealed a modal diameter of 91 nm (range: 24–295 nm; D10 = 51 nm; D90 = 190 nm), with a slight increase in diameter upon binding to the SpoVM‐c‐CPE^Q317I^ peptide (modal: 142 nm; range: 24–459 nm; D10 = 58.7 nm; D90 = 255 nm) (Figure 2i). With the help of the mutated peptide SpoVM‐c‐CPE^Q317I^, confocal imaging showed stronger adhesion of NK‐sEVs to the surface of GC compared with NK‐sEVs binding to wild‐type SpoVM‐c‐CPE peptides (Figure 2j,k). Meanwhile, we observed that the Q317I conferred enhanced binding affinity of NK‐sEVs compared with Q317M. Taken together, we successfully designed and acquired a novel NESC complex capable of targeting GC cells.

### 3.3 NK‐sEVs Function as a ROS Inducer and Radio‐Sensitiser

**FIGURE 3:**
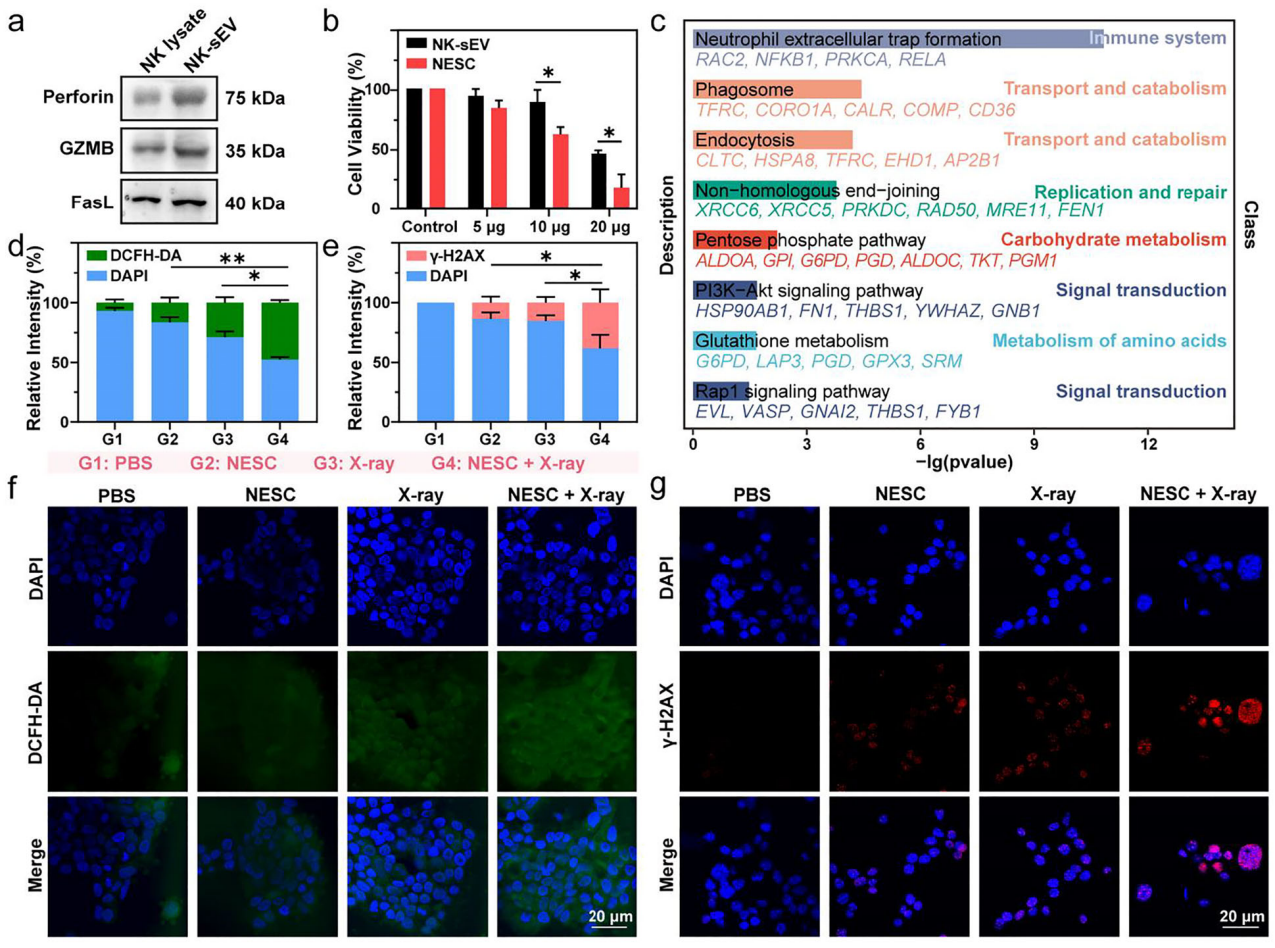
ROS induction and radiosensitising effects of NESC in NCI‐N87 cells. (a) Western blot analysis of perforin, granzyme B and FasL in NK cell lysate and NK‐sEV. (b) Cell viability of NCI‐N87 cells incubated with three concentrations of NK‐sEVs or NESC (5, 10 and 20 µg). Viability of PBS‐treated cells was considered as a base value. Data was shown as mean ± s.d. (*n* = 3). (c) Bar plot showing the KEGG enrichment analysis results, including the pathway names, related genes and pathway classifications. (d and f) Confocal images (f) and quantified data (d) of ROS generation in NCI‐N87 cells treated with different regimens. (blue: DAPI, green: DCFH‐DA). (e and g) Confocal images (g) and quantified data (e) of DNA breaks in NCI‐N87 cells treated with different regimens. (blue: DAPI, red: γ‐H2AX). G1: PBS, G2: NESC (200 µg/mL), G3: 2 Gy and G4: NESC (200 µg/mL) + 2 Gy. *p* values were calculated by an unpaired *t*‐test (*p < 0.05, **p < 0.01).

NK cells are widely known to exert cytotoxic effects on target cells in the form of lytic granules (Coënon et al. 2024). Interestingly, we found that NK‐sEVs exhibited higher levels of perforin, granzyme B and FasL than those of NK cell lysates under the same protein quantification, suggesting that NK‐sEVs might be more cytotoxic than NK cells (Figure 3a). NCI‐N87 cells were incubated with different concentrations of NK‐sEVs for 24 h, either with or without SpoVM‐c‐CPE^Q317I^ conjugation. Cell counting kit‐8 (CCK‐8) assay showed that both inhibited cell viability in a dose‐dependent manner (5, 10 and 20 µg per well in 96‐well plates) and NESC exhibited significantly stronger cytotoxicity than NK‐sEVs alone (Figure 3b). To further explore the predominant antitumour mechanism, liquid chromatography‐mass spectrometry (LC‐MS) was conducted, and a total of 963 proteins were identified (Table S3). After trimming off possible protein contaminants, KEGG enrichment analysis was performed (Figure 3c). Among these proteins, XRCC5, XRCC6, RAD50 and MRE11 were enriched in the pathway termed non‐homologous end‐joining, which was classified into the DNA replication and repair group, suggesting that NK‐sEVs might play a role as a radiotherapy sensitiser.

Additionally, the pentose phosphate pathway and glutathione metabolism pathway were also statistically significant, which were involved in carbohydrate metabolism and amino acid metabolism, respectively. These metabolic activities would generate free electrons, activate the electron transfer chain and then generate ROS. It was reported that ROS could induce DNA damage and affect the DNA damage response, suggesting that ROS modulators could be used to enhance radiotherapy response (Srinivas et al. 2019; Juan et al. 2021; Lin and Epel 2022).

Enhancement of ROS production by NESC was further validated by confocal microscopy. When NCI‐N87 cells were treated with NESC (200 µg/mL) or x‐rays (2 Gy) alone, higher ROS levels were observed than in the PBS‐treated group. Surprisingly, when NESC (200 µg/mL) and x‐rays (2 Gy) were applied simultaneously, an even higher fluorescence intensity of ROS production was observed (Figure 3d,f). This was consistent with the aforementioned pathway enrichment results and implied that NESC might act as a radio‐sensitiser by increasing ROS. In order to validate the potential radio‐sensitisation effect of NESC, γ‐H2AX immunofluorescence staining was carried out to detect DNA double‐strand breaks in GC cells. NCI‐N87 cells were seeded in cell culture plates and then treated with NESC (200 µg/mL), x‐rays (2 Gy) or NESC (200 µg/mL) plus x‐rays (2 Gy). NCI‐N87 cells treated with PBS were used as a control group. The results showed that cancer cells treated with NESC alone showed limited effect on DNA structural damage, while the fluorescence intensity of cells treated with the combination of NESC and x‐rays was significantly stronger than that of other groups (Figure 3e,g). Collectively, these findings demonstrated that NESC not only exhibited potent cytotoxicity but also enhanced radiosensitivity in GC cells through ROS‐mediated DNA damage potentiation.

### 3.4 GC Organoids Reveal the Antitumour Potential of NESC

**FIGURE 4:**
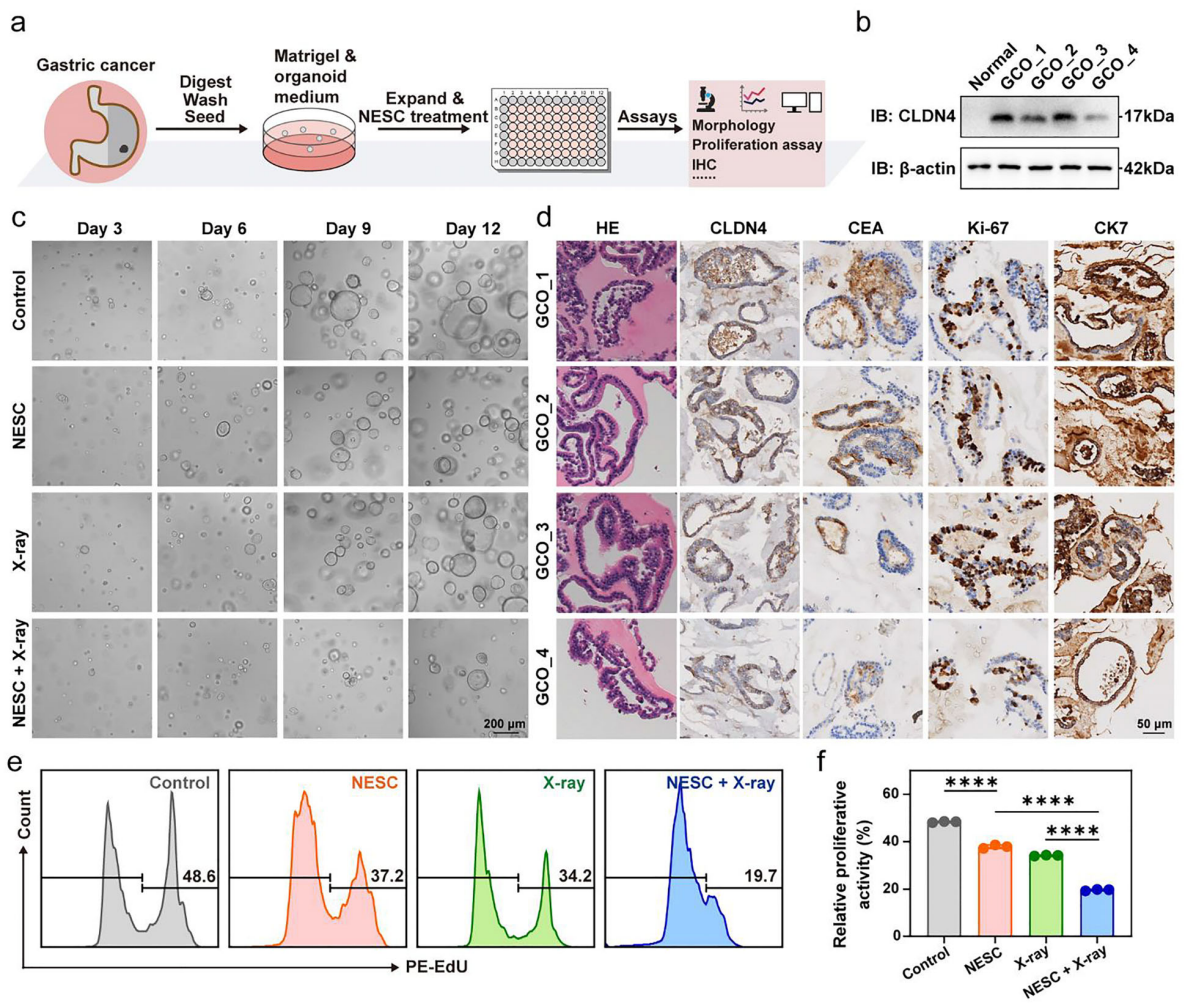
GC patient‐derived organoids treated with NESC. (a) Schematic illustration of GCO experiment procedures. (b) Western blot analysis of CLDN4 expression in normal gastric organoids and GC organoids. (c) Bright field microscopy of GCOs in each treatment group. (d) H&E and IHC staining of CLDN4, CEA, Ki‐67 and CK7 in GCOs. (e and f) Flow cytometry results showing degrees of proliferative activity of organoids in each group (e) and the corresponding statistical plot (f) (*n* = 3). G1: PBS, G2: NESC (200 µg/mL), G3: 2 Gy and G4: NESC (200 µg/mL) + 2 Gy. *p* values were calculated by multiple *t*‐tests (****p < 0.0001).

Patient‐derived organoids (PDOs) display diverse morphological phenotypes that correlate with their underlying genotypes, including cystic structures, irregular shapes, wrinkled cell layers, ‘grape‐like’ appearance and so forth (Wallaschek et al. 2019). In this study, histologically diagnosed GC tissues were obtained from surgically resected specimens and four GC organoid (GCO) lines were established according to the published protocols (Figure 4a) (Yan et al. 2018). Compared with the normal gastric organoids, all four GCO lines exhibited elevated CLDN4 expression (Figure 4b). Bright‐field microscopy revealed that GCOs untreated with NESC maintained a vesicular three‐dimensional structure with a monolayer epithelial lining after multiple passages (Figure 4c, Control). Histopathological evaluation via hematoxylin and eosin (H&E) staining and immunohistochemical (IHC) analysis confirmed the expression of established GC biomarkers, including CEA, Ki‐67, and CK7. Consistent with immunoblotting data, these organoids exhibited prominent CLDN4 expression (Figure 4d). Over time, control GCOs progressively increased in size and cellular density, displayed darker pigmentation, and developed thickened walls (Figure 4c, Control). In striking contrast, NESC‐treated (200 µg/mL) GCOs underwent significant morphological alterations, losing their spherical integrity in bright‐field images. The organoid walls appeared shrivelled and fragmented, with indistinct boundaries between adjacent structures (Figure 4c, NESC). To investigate the radiosensitising effect of NESC on GCOs, we treated GCOs with a combination of NESC (200 µg/mL) and x‐ray irradiation (2 Gy) (Figure 4c, NESC + x‐ray). The proliferative activity of GCOs with different treatments was analysed by an EdU proliferation assay. Pretreatment with NESC prior to x‐ray irradiation resulted in impaired GCO proliferation compared with either treatment alone (Figure 4e,f). These findings provided compelling evidence for the therapeutic value of NESC at the organoid level, highlighting its dual role as both an antitumour agent and radiation sensitiser in GC models.

### 3.5 Multimodal Imaging Confirms Tumour‐Specific NESC Localisation and Enhanced Therapeutic Efficacy in GC

**FIGURE 5:**
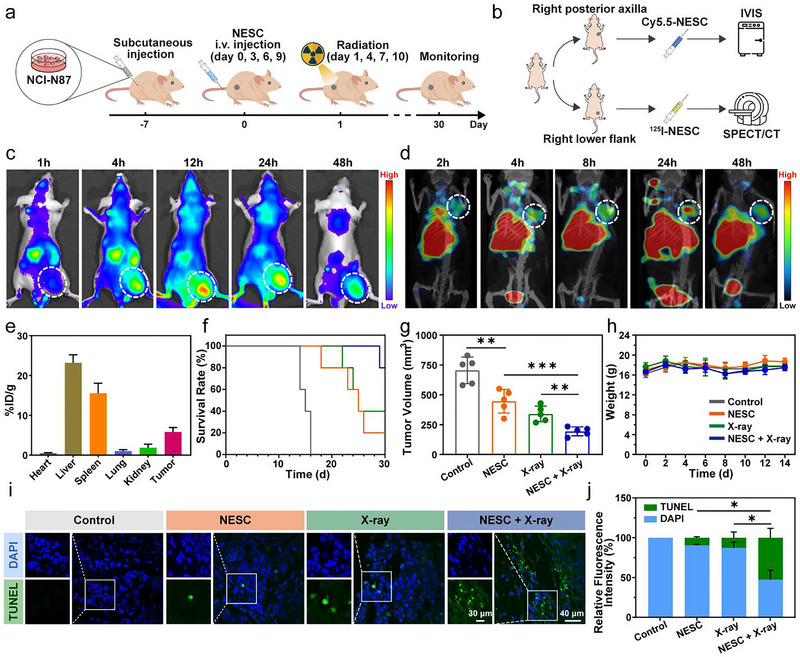
Targeted delivery of NESC to subcutaneous GC tumour. (a) Schematic illustration of nude mice receiving NESC and/or x‐ray. (b) Schematic illustration of tumour‐bearing nude mice examined by IVIS and SPECT/CT. (c) Biodistribution of Cy5.5‐labelled NESC monitored by IVIS at different time points. (d and e) Representative SPECT/CT images showing biodistribution of ^125^I‐labelled NESC at different time points (d) and the quantified data (e) at 48 h. (f) Survival rate of mice after different treatments. (g) Tumour volumes of nude mice in each treatment group on Day 14. (h) Body weight changes of mice post‐treatments. (i and j) Confocal images (i) and quantified data (j) of TUNEL staining of tumour slices collected from mice in each treatment group (blue: DAPI, green: TUNEL). Data were shown as mean ± s.d. *n* = 5 in (e)–(h). G1: control, G2: NESC (100 µg × 4), G3: 2 Gy × 4 and G4: NESC (100 µg × 4) + 2 Gy × 4. *p* values were calculated by multiple *t*‐tests (*p < 0.05, **p < 0.01, ***p<0.001).

To evaluate the therapeutic potential of NESC in vivo, we established an NCI‐N87 GC xenograft model in BALB/c nude mice. NCI‐N87 cells were implanted subcutaneously in the right posterior axillae or lower flanks, and when tumours reached approximately 100 mm^3^ (∼1 week post‐implantation), mice were randomized into four treatment groups (*n* = 5 per group): G1: control, G2: NESC (100 µg × 4), G3: 2 Gy × 4 and G4: NESC (100 µg × 4) + 2 Gy × 4. G4 received intravenous NESC (100 µg) followed by localised x‐ray irradiation (2 Gy) after 24 h, with this cycle repeated four times (Figure 5a **)**. Prior to the application of NESC to treat tumour‐bearing mice, its biodistribution was first monitored by IVIS and SPECT/CT at various time points after i.v. injection (Figure 5b). The results showed that Cy5.5‐labelled NESC significantly accumulated in the tumour site after 12 h and remained in the tumour site until 48 h (Figure 5c). Afterwards, major organs including heart, liver, spleen, lung, kidney and tumour were collected and imaged by IVIS (Figure S7a). The tumour tissue exhibited a stronger fluorescent signal compared with other tissues (Figure S7b). Meanwhile, SPECT/CT tracking of ^125^I‐labeled NESC demonstrated detectable tumour signals as early as 2 h post‐injection, peaking at 24 h (Figure 5d,e).

Therapeutic outcomes revealed striking differences: PBS‐treated controls succumbed rapidly to cancer cachexia or required euthanasia due to excessive tumour burden, whereas the combination group showed maximal survival extension (Figure 5f). Surprisingly, NESC in combination with x‐rays significantly inhibited the tumour growth compared with the other groups, suggesting that the specially designed NESC had a synergistic effect with radiotherapy (Figure 5g and Figure S7c,d). Moreover, there were no significant differences in the body weights of the mice with different treatments (Figure 5h). Meanwhile, TdT‐mediated dUTP Nick‐End Labelling (TUNEL) staining also verified that injection of NESC before x‐ray irradiation induced the most severe DNA damage (Figure 5i,j). Therefore, these findings demonstrated that NESC exhibited: (i) excellent tumour‐selective targeting, (ii) a favourable biosafety profile and (iii) potent radiosensitising effects, establishing its promise as a novel therapeutic agent for GC.

### 3.6 NESC Synergises With Radiotherapy to Inhibit GC in the Patient‐Derived Organoid Xenograft (PDOX) Model

**FIGURE 6:**
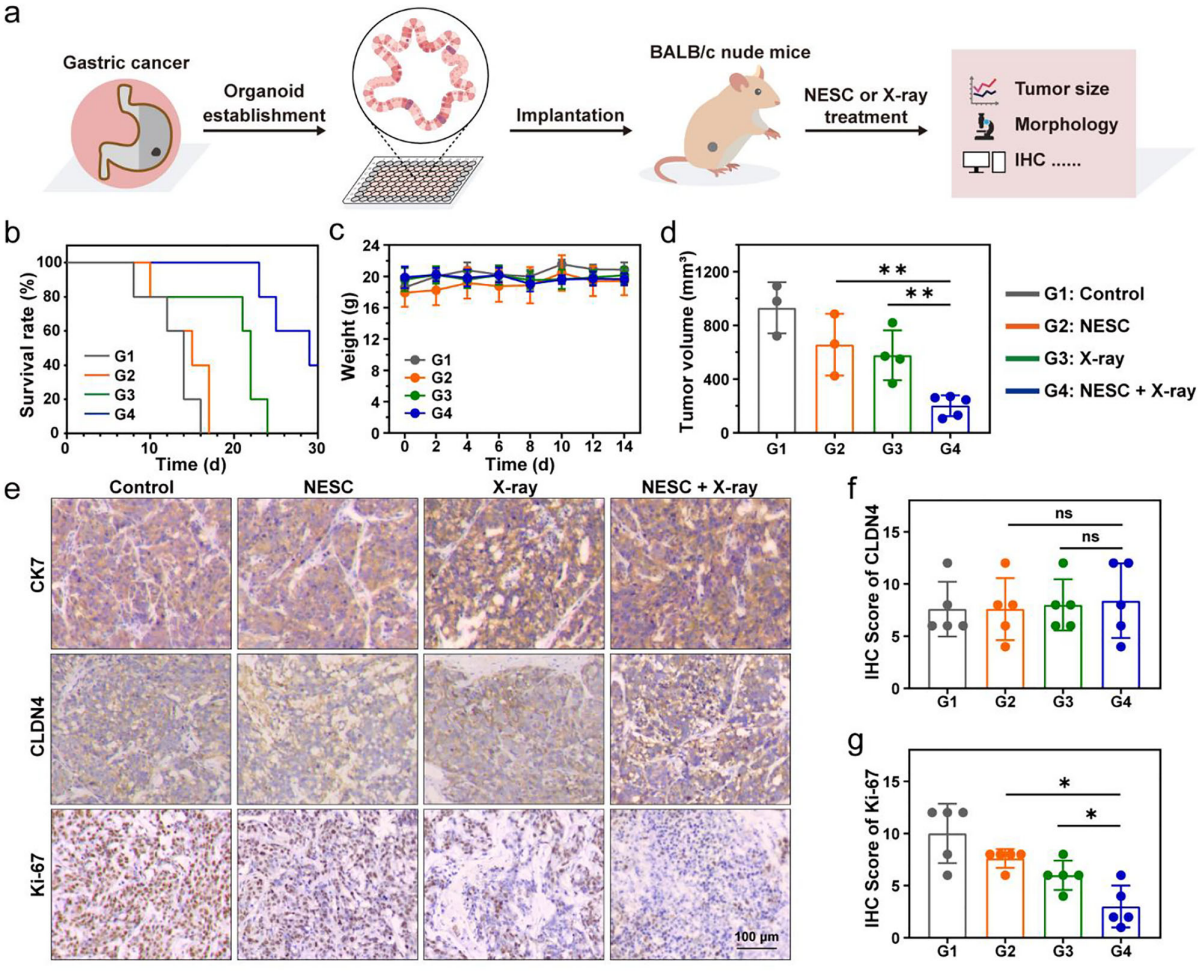
Validation of the radiosensitising effect of NESC using a GC PDOX model. (a) Schematic illustration depicting the establishment of the GC PDOX model. (b) Survival rate of PDOX‐bearing mice after different treatments. (c) Body weight changes of PDOX‐bearing mice after different treatments. (d) Tumour volumes of nude mice in each treatment group on Day 14. (e) IHC staining of CK7, CLDN4 and Ki‐67 of tumour tissues in each treatment group. (f and g) Barplots showing scores of IHC staining of CLDN4 (f) and Ki‐67 (g) of tumour tissues in each treatment group*. n* = 5 in (b), (c), (f) and (g). Sample sizes were G1 (*n* = 3), G2 (*n* = 3), G3 (*n* = 4) and G4 (*n* = 5) in (d). G1: control, G2: NESC (100 µg × 4), G3: 2 Gy × 4 and G4: NESC (100 µg × 4) + 2 Gy × 4. *p* values were calculated by multiple *t*‐tests (*p < 0.05, **p < 0.01).

To further validate the radiosensitising effect of NESC in vivo, we established a patient‐derived organoid xenograft (PDOX) model (Figure 6a). The abovementioned organoids were expanded and subcutaneously implanted into the right lower flank of nude mice. Then these mice were randomly divided into four groups (*n* = 5 per group): G1: control, G2: NESC (100 µg × 4), G3: 2 Gy × 4 and G4: NESC (100 µg × 4) + 2 Gy × 4. Survival analysis demonstrated that the combination of NESC with x‐ray irradiation significantly prolonged mouse survival, with 40% of animals (*n* = 2) surviving until the end of the observation period (Figure 6b). Notably, all treatments were well‐tolerated, as evidenced by stable body weights across all groups without significant intergroup differences (Figure 6c). The tumour volume was also monitored during the experiment. All tumours in the combination group had volumes below 400 mm^3^ at the end of the 2‐week observation period, which were significantly smaller than those in the other groups (Figure 6d and Figure S8a,b). After the observation, tumours were harvested from each group, and IHC of CK7, CLDN4 and Ki‐67 was conducted. All groups showed high expression levels of CK7 and CLDN4, confirming the epithelial origin and the potential to be targeted by NESC of the tumours (Figure 6e). In contrast, the expression of the proliferation marker Ki‐67 showed a marked reduction, with the most pronounced decrease observed in the combination group, indicating enhanced antiproliferative efficacy of the combined NESC and radiotherapy regimen (Figure 6e,g). These findings collectively demonstrate that NESC acted as a potent radiosensitizer PDOX model, significantly enhancing the therapeutic efficacy of radiotherapy while maintaining a favourable safety profile in vivo.

## 4 Discussion

GC is one of the leading causes of cancer‐related mortality worldwide (Sung et al. 2021). Given the heterogeneity in molecular features among GC patients, the identification of novel therapeutic targets is urgently needed. In recent years, the CLDN family has emerged as a promising focus in cancer research. Under physiological conditions, CLDNs are largely concealed within normal tissues due to their localisation at the apical component of cell‐cell junctions. However, in tumour tissues, the disruption of tight junction integrity leads to CLDN exposure, rendering them accessible for therapeutic targeting (Bang et al. 2023). Among CLDN family members, claudin‐18 isoform 2 (CLDN18.2) has garnered significant attention, particularly with the ongoing phase 3 GLOW (Shah et al. 2023) and phase 3 SPOTLIGHT trials (Shitara et al. 2023) evaluating zolbetuximab—a CLDN18.2‐targeting monoclonal antibody—in combination with capecitabine and oxaliplatin (CAPOX) or modified FOLFOX6 (mFOLFOX6). In contrast, CLDN4 has long been recognised as a critical player in cancer due to its frequent upregulation in multiple malignancies. Our current study, through single‐cell RNA sequencing (scRNA‐seq), tissue microarray analysis, and Western blot, confirmed CLDN4 overexpression in patient‐derived GC tissues. Previous reports by Ma et al. suggested that CLDN4 may act as a downstream effector of TSPEAR‐AS2 in GC progression (Ma et al. 2020). However, the functional role of CLDN4 in GC remains debated. Kwon et al. demonstrated that elevated membranous CLDN4 suppresses GC cell migration and invasion by reinforcing tight junction barrier function (Kwon et al. 2011). Luo et al. reported that CLDN4 enhances GC cell chemosensitivity by inhibiting the PI3K/AKT signalling pathway (Luo et al. 2020). Despite these conflicting findings, CLDN4 remains a promising therapeutic target for GC patients exhibiting high CLDN4 expression.

Nowadays, antibody‐drug conjugates (ADCs) have garnered increasing attention in recent years as a promising therapeutic strategy. A typical ADC comprises three crucial elements: (1) an antibody that selectively targets cancer cells while potentially eliciting a limited therapeutic response, (2) a cytotoxic payload responsible for the primary antitumour effects and (3) a linker that conjugates the antibody to the payload (Wang et al. 2023). Inspired by this design, we developed an innovative alternative by replacing the antibody with a targeting peptide and utilising human‐derived EVs as the payload. This approach offers several advantages: (1) the significantly lower molecular weight of peptides compared to antibodies allows for higher EV conjugation density, (2) EVs can efficiently deliver their cargo into target cells through direct membrane fusion and (3) peptides exhibit superior biodegradability with minimal risk of adverse effects. However, these theoretical advantages require further experimental validation. The N‐terminus of our engineered peptide incorporates SpoVM, a 26‐amino‐acid protein derived from Bacillus subtilis that plays a crucial role in sporulation (Levin et al. 1993). During bacterial spore formation, SpoVM selectively accumulates on the convex curvature of the forespore membrane and subsequently recruits additional structural proteins (Kim et al. 2017; Peluso et al. 2019). Capitalising on this unique property, we designed an EV surface display system to effectively coat NK cell‐derived small extracellular vesicles (NK‐sEV) with the SpoVM‐c‐CPE^Q317I^ conjugate.

Organoids represent an advanced three‐dimensional cell culture system derived from differentiated normal or tumour tissues (Xu et al. 2018). These structures preserve the genomic stability and tumour heterogeneity of their tissue of origin, making them invaluable tools for disease modelling, drug response evaluation, and personalised treatment development (Pang et al. 2021). In this study, we designed a novel sEV–peptide conjugate termed NK‐sEV‐SpoVM‐c‐CPE^Q317I^ (NESC), composed of NK‐92MI cell‐derived small extracellular vesicles and a mutant CLDN4‐targeting peptide (SpoVM‐c‐CPE^Q317I^), for selective targeting of GC cells overexpressing CLDN4. NESC not only exhibited direct cytotoxicity against GC cells but also elevated intracellular ROS levels, thereby enhancing the efficacy of radiotherapy. The targeting specificity and therapeutic potential of NESC were systematically validated in multiple experimental models, including GC cell lines, patient‐derived organoids (PDO), and patient‐derived orthotopic xenograft (PDOX) mouse models.

## 5 Conclusion

In light of our discovery of elevated CLDN4 expression in GC using single‐cell transcriptomics, we devised a novel therapeutic agent termed NESC to specifically target CLDN4‐overexpressing GC cells, aiming to bolster the effectiveness of GC treatment. These innovative biomaterials incorporated a meticulously designed peptide, c‐CPE^Q317I^, which could bind tightly to the membrane curvature‐sensing SpoVM peptide, ensuring stable attachment to NK‐sEVs and yielding NESC. Our experiments conclusively demonstrated that NESC effectively invaded GC cells and exerted potent cytotoxic effects on GC cell lines, patient‐derived GC organoids, and GC tumours in mice. Notably, NESC significantly elevated intracellular ROS levels, thereby amplifying the therapeutic impact of radiotherapy. Therefore, our work demonstrated for the first time the efficacy of targeting NESC in combination with radiotherapy, providing a new avenue for precision treatment of GC.

## Author Contributions

**Anqi Dong**: writing–original draft, software. **Wenhao Shen**: investigation, methodology. **Xiaochun Shen**: software. **Shu Liu**: methodology. **Dongbao Li**: methodology. **Min Li**: methodology. **Minghui Li**: methodology. **Yan Ma**: methodology. **Jin Zhou**: conceptualization. **Lin Hu**: conceptualization. **Kai Yang**: conceptualization, resources, project administration, writing–review and editing.

## Funding

This work was partially supported by the National Natural Science Foundation of China (32171382, 82372887), Key Research and Development Program of Social Development of Jiangsu Province (BE2022725), the Project Fund by the Priority Academic Program Development of Jiangsu Higher Education Institutions (PAPD), Suzhou Fundamental Research Project (SJC2023001), the Natural Science Foundation of Jiangsu Province (BK20221242), Gusu Health Talents Cultivation Program, China (GSWS2019008), the “Qinglan Project” in Jiangsu Colleges and Universities, the Special Project of Diagnosis and Treatment Technology for Clinical Key Diseases in Suzhou (LCZX202202), the Clinical Medicine Peak Project of Suzhou Medical College of Soochow University, and the leading talent project of “Boxi Excellent Talent Training Plan” of the First Affiliated Hospital of Soochow University.

## Conflicts of Interest

The authors declare no conflicts of interest.

## Supporting information




**Supplementary Material**: jev270200‐sup‐0001‐SuppMat.docx

## Data Availability

Data sharing is not applicable to this article as no datasets were generated or analysed during the current study.
