# Supplementary material for: NK Cell‐Derived Small Extracellular Vesicles Armed With CLDN4‐Targeting Peptides Potentiate Radiotherapy in Gastric Cancer
Source: J Extracell Vesicles. 2025 Nov 18;14(11):e70200. doi: 10.1002/jev2.70200 (PMC12626167; doi:10.1002/jev2.70200)
Supplement: Supplementary file 1 — Supplementary Material: jev270200‐sup‐0001‐SuppMat.docx [file JEV2-14-e70200-s001.docx]

SUPPLEMENTAL INFORMATION

NK Cell-Derived Small Extracellular Vesicles Armed with CLDN4-Targeting Peptides Potentiate Radiotherapy in Gastric Cancer

Anqi Dong^1,#^, Wenhao Shen^3,#^, Xiaochun Shen^4,#^, Shu Liu^1^, Dongbao Li^2^, Min Li^5^, Minghui Li^6^, Yan Ma^7^, Jin Zhou^2,^*, Lin Hu^1,^*, Kai Yang^1,^*

1. Department of Pathology, the First Affiliated Hospital, State Key Laboratory of Radiation Medicine and Protection, School of Radiation Medicine and Protection & School for Radiological and Interdisciplinary Sciences (RAD-X), Collaborative Innovation Center of Radiation Medicine of Jiangsu Higher Education Institutions, Cancer Institute, Suzhou Medical College, Soochow University, Suzhou 215123, Jiangsu, China.

2. Department of General Surgery, the First Affiliated Hospital of Soochow University, Soochow University, Suzhou 215006, Jiangsu, China.

3. Department of Central Laboratory and Oncology, Taizhou People's Hospital Affiliated to Nanjing Medical University, Taizhou 225300, Jiangsu, China.

4. Department of Respiratory, the First Affiliated Hospital of Soochow University, Soochow University, Suzhou 215006, Jiangsu, China.

5 Institutes of Biology and Medical Sciences, Soochow University, Suzhou 215123, Jiangsu, China.

6. MOE Key Laboratory of Geriatric Diseases and Immunology, School of Biology and Basic Medical Sciences, Soochow University, Suzhou 215123, Jiangsu, China.

7. Department of Pathology, the First Affiliated Hospital of Soochow University, Soochow University, Suzhou 215006, Jiangsu, China.

^#^ These authors contributed equally to this work.

* Correspondence: hulin@suda.edu.cn, zhoujinsuda@suda.edu.cn, kyang@suda.edu.cn

**Supporting figures**

**
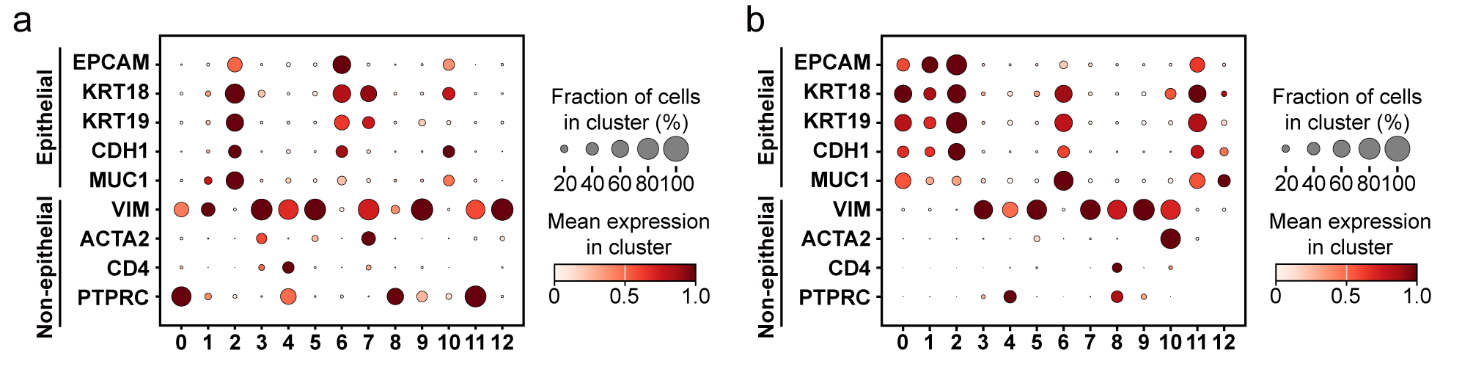
**

**Supplementary Fig. S1. Dot plots of specific marker genes in GSE183904 and GSE134520. a,b** Dot plots depicting expression of epithelial (EPCAM, KRT18, KRT19, CDH1 and MUC1) and non-epithelial (VIM, ACTA2, CD4 and PTPRC) marker genes together with the percentage of cells expressing the marker in GSE183904 (**a**) and GSE134520 (**b**).

**
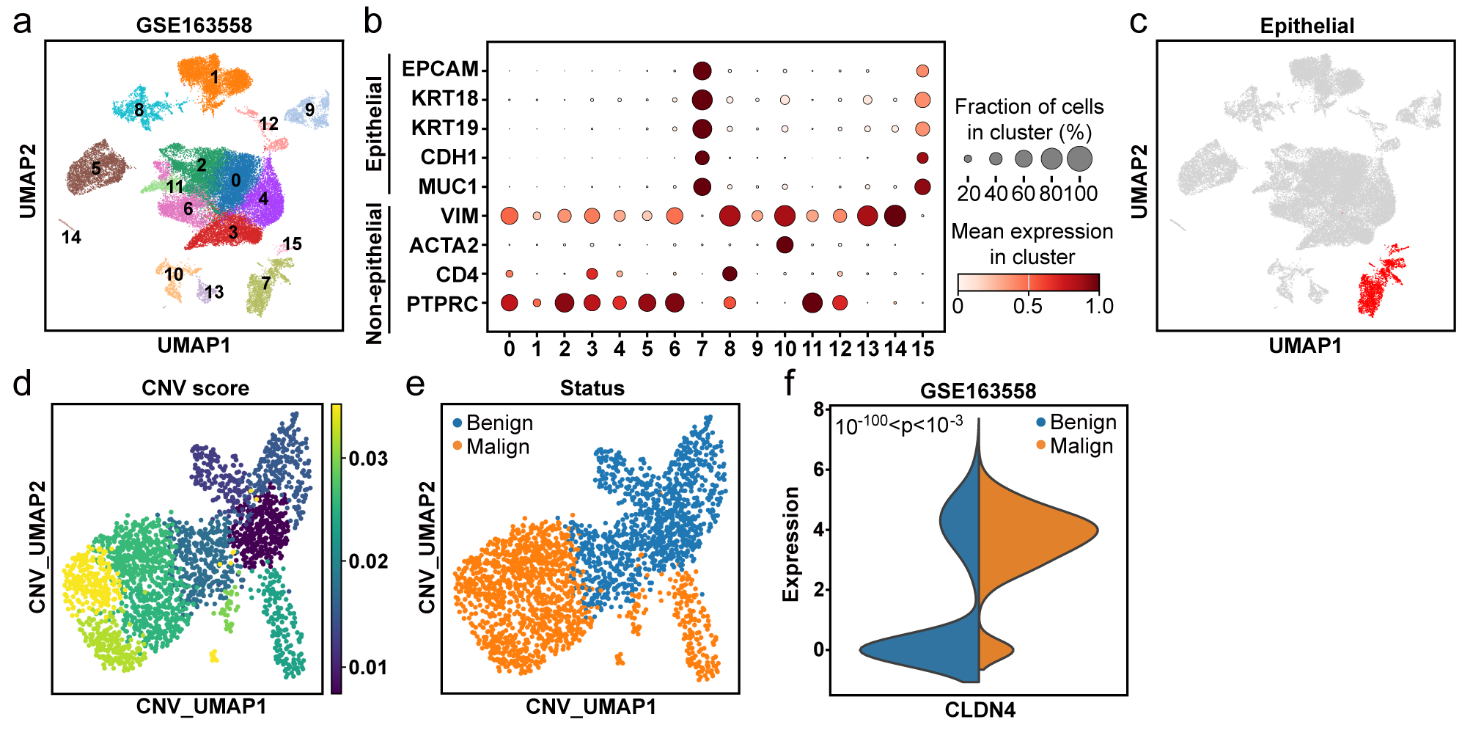
**

**Supplementary Fig. S2. In silico analysis of CLDN4 expression in GSE163558. a,** UMAP plot of all cells in GSE163558 colored by cluster identity. **b,** Dot plot depicting expression of epithelial (EPCAM, KRT18, KRT19, CDH1 and MUC1) and non-epithelial (VIM, ACTA2, CD4 and PTPRC) marker genes together with the percentage of cells expressing the marker in GSE163558. **c,** UMAP plot of GSE163558 highlighting epithelial cells (red) versus non-epithelial cells (gray). **d,** UMAP plot depicting the CNV scores of all epithelial cells in GSE163558. **e,** UMAP plot showing the classification of malignant and non-malignant cells in GSE163558. **f,** Half violin plot showing CLDN4 expression in putative malignant and non-malignant cells in GSE163558.

**
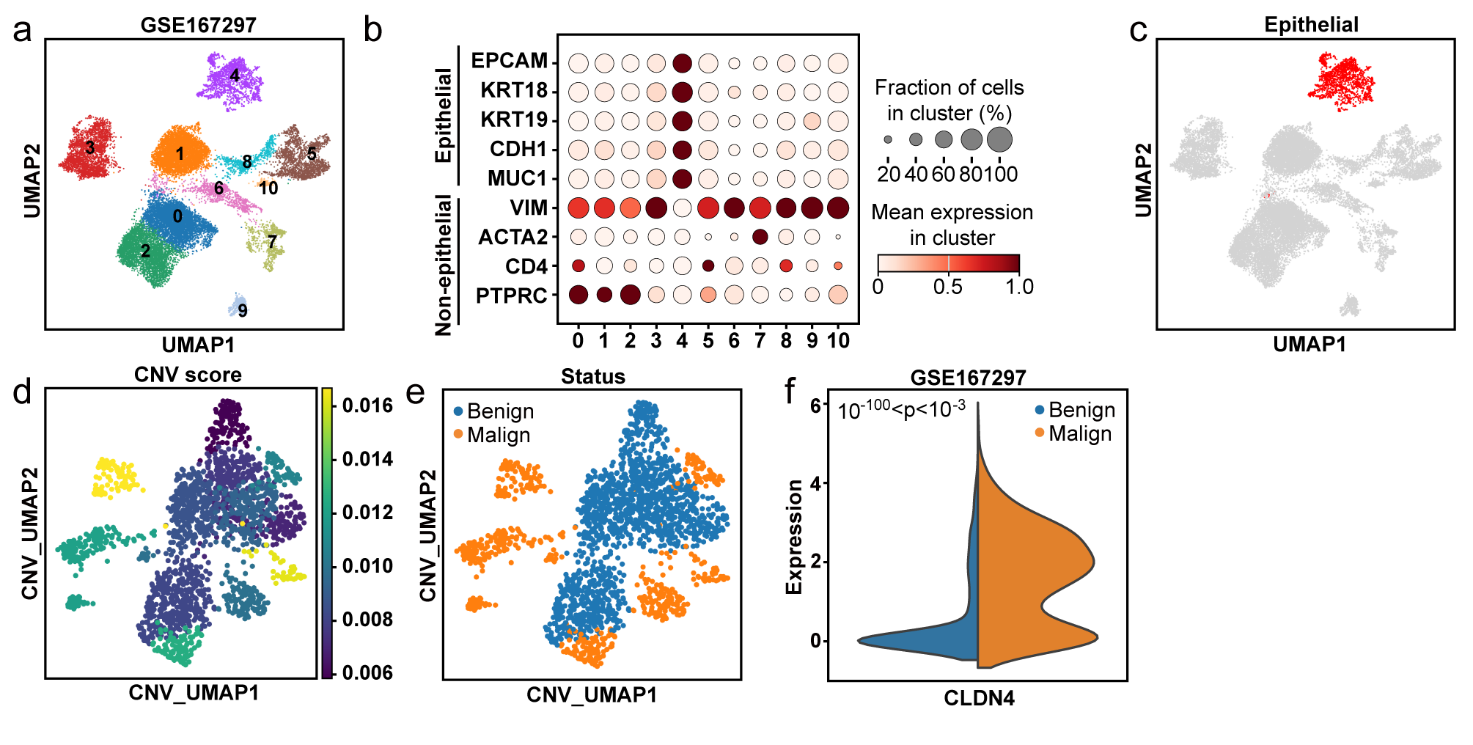
**

**Supplementary Fig. S3. In silico analysis of CLDN4 expression in GSE167297. a,** UMAP plot of all cells in GSE167297 colored by cluster identity. **b,** Dot plot depicting expression of epithelial (EPCAM, KRT18, KRT19, CDH1 and MUC1) and non-epithelial (VIM, ACTA2, CD4 and PTPRC) marker genes together with the percentage of cells expressing the marker in GSE167297. **c,** UMAP plot of GSE167297 highlighting epithelial cells (red) versus non-epithelial cells (gray). **d,** UMAP plot depicting the CNV scores of all epithelial cells in GSE167297. **e,** UMAP plot showing the classification of malignant and non-malignant cells in GSE167297. **f,** Half violin plot showing CLDN4 expression in putative malignant and non-malignant cells in GSE167297.

**
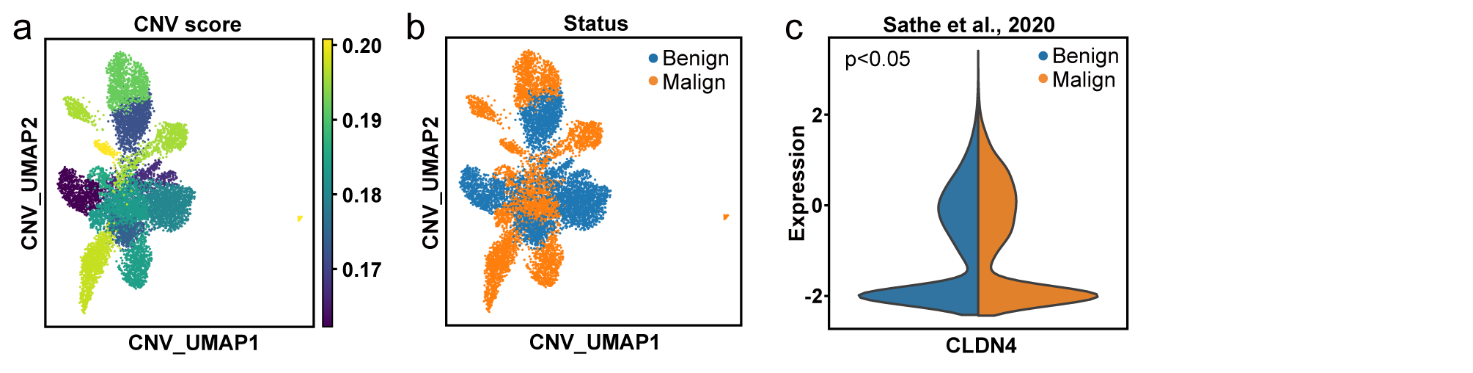
**

**Supplementary Fig. S4. In silico analysis of CLDN4 expression in datasets provided by Sathe and colleagues. a,** UMAP plot depicting the CNV scores of all epithelial cells. **b,** UMAP plot showing the classification of malignant and non-malignant cells. **c,** Half violin plot showing CLDN4 expression in putative malignant and non-malignant cells.


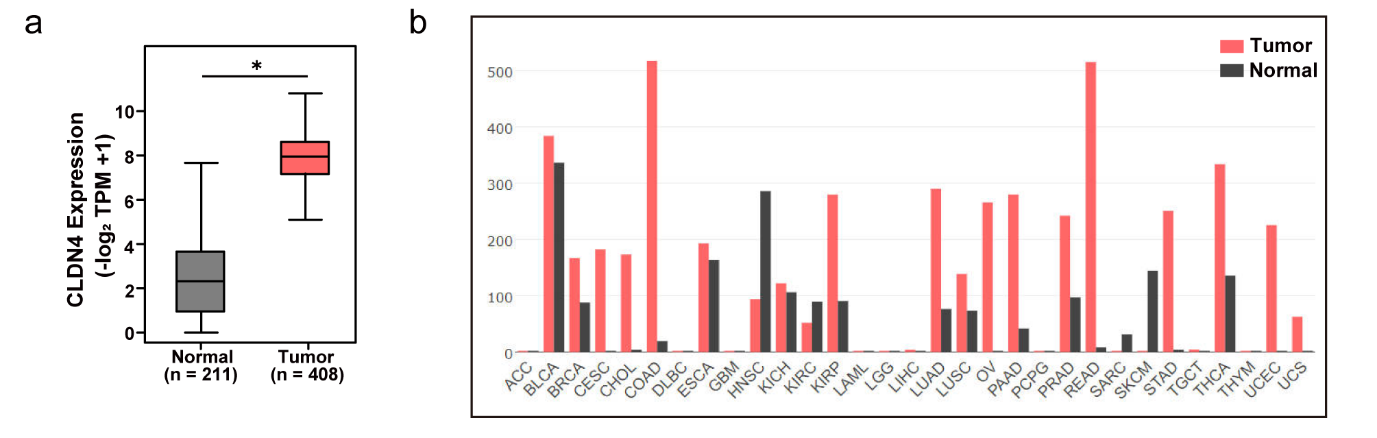


**Supplementary Fig. S5. Analysis of CLDN4 expression in TCGA cohorts. a,** Box plot showing the difference of CLDN4 expression in normal and tumor tissues from TCGA. **b,** CLDN4 expression in all cancer types in TCGA. (**p* < 0.05)


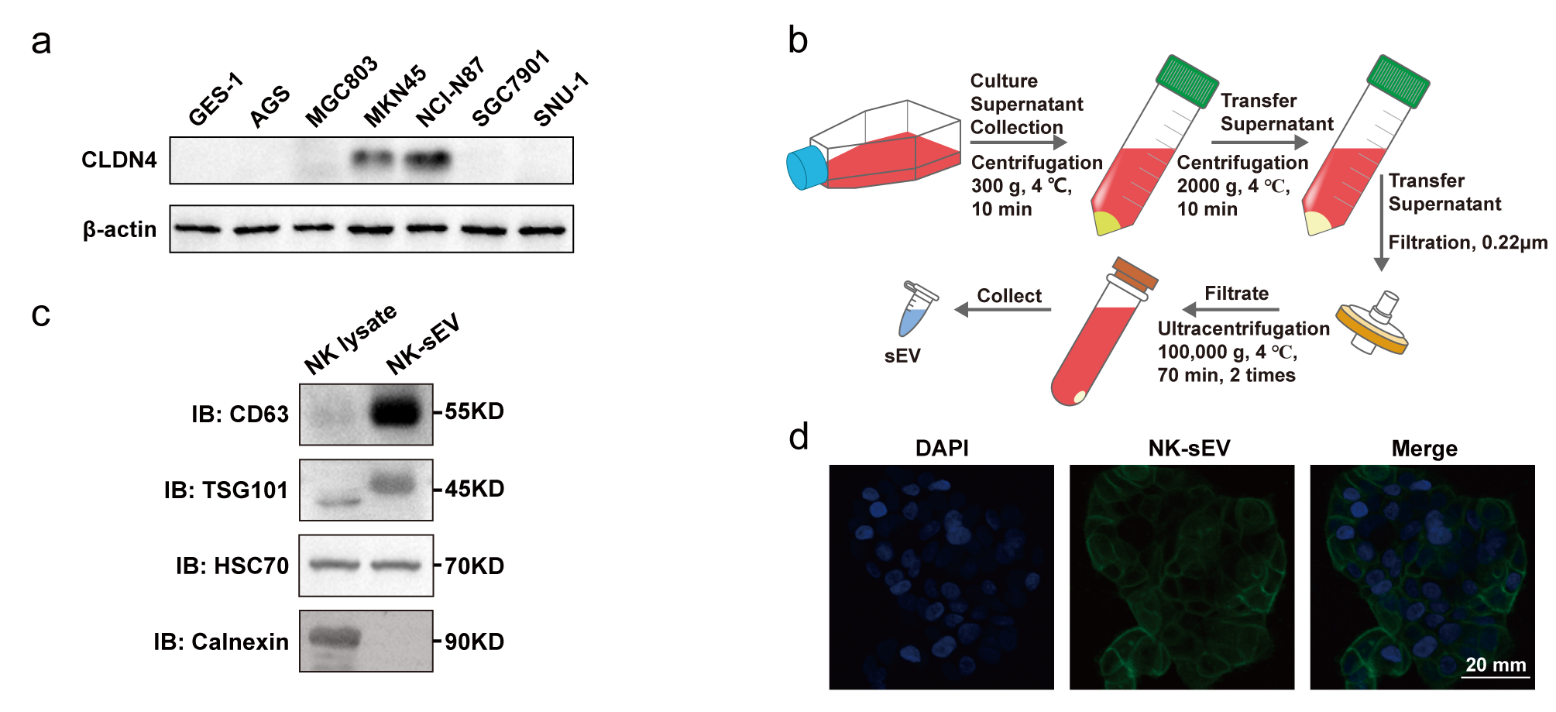


**Supplementary Fig. S6. Isolation and characteristics of NK-sEVs. a,** Western blot analysis of CLDN4 expression in GC cell lines. **b,** Schematic illustration of sEV isolation by ultracentrifugation. **c,** Western blot analysis showing the expression of EV marker proteins (CD63, TSG101 and HSC70) and a negative marker (Calnexin). **d,** Representative confocal images of NK-sEV accumulation on NCI-N87 cell membrane (blue: DAPI, green: NK-sEV).


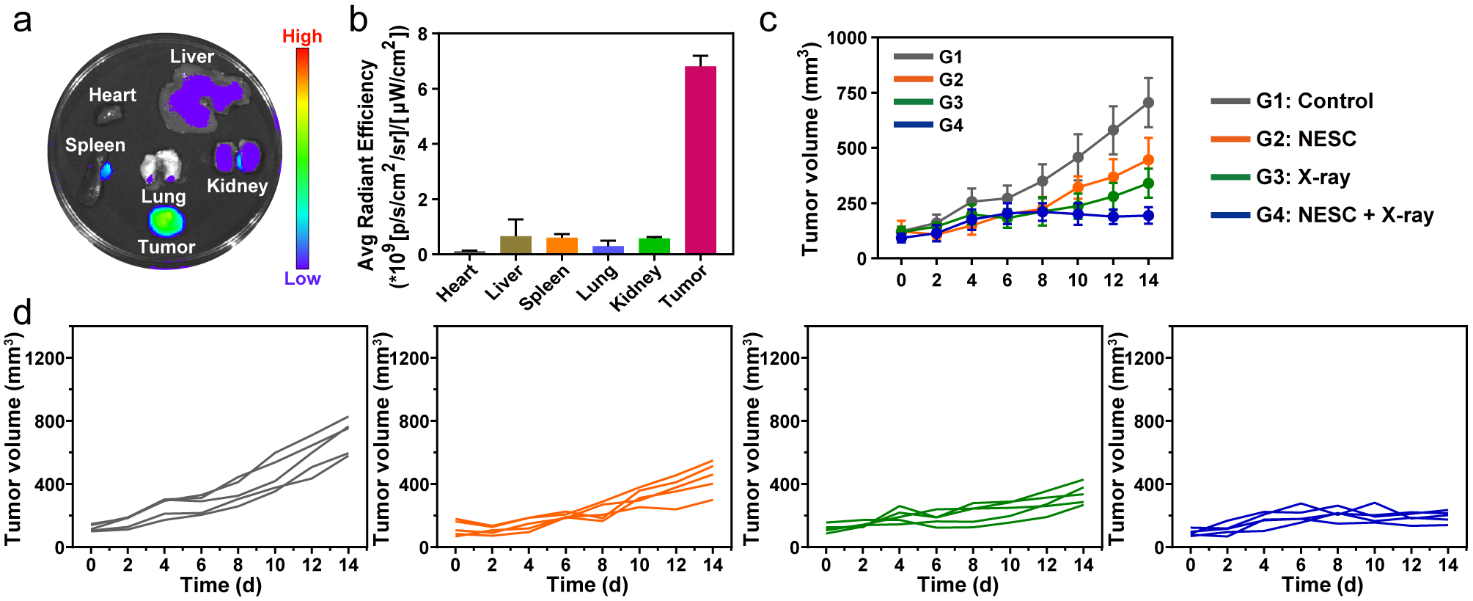


**Supplementary Fig. S7. Biodistribution and antitumor effect of NESC. a,b,** Fluorescence image of the main organs collected from mice 48 h post administration (**a**) and its intensity statistical plot (**b**). **c,d,** Respective tumor growth curves of each group (**c**) and each mouse (**d**) during 14 days.

**
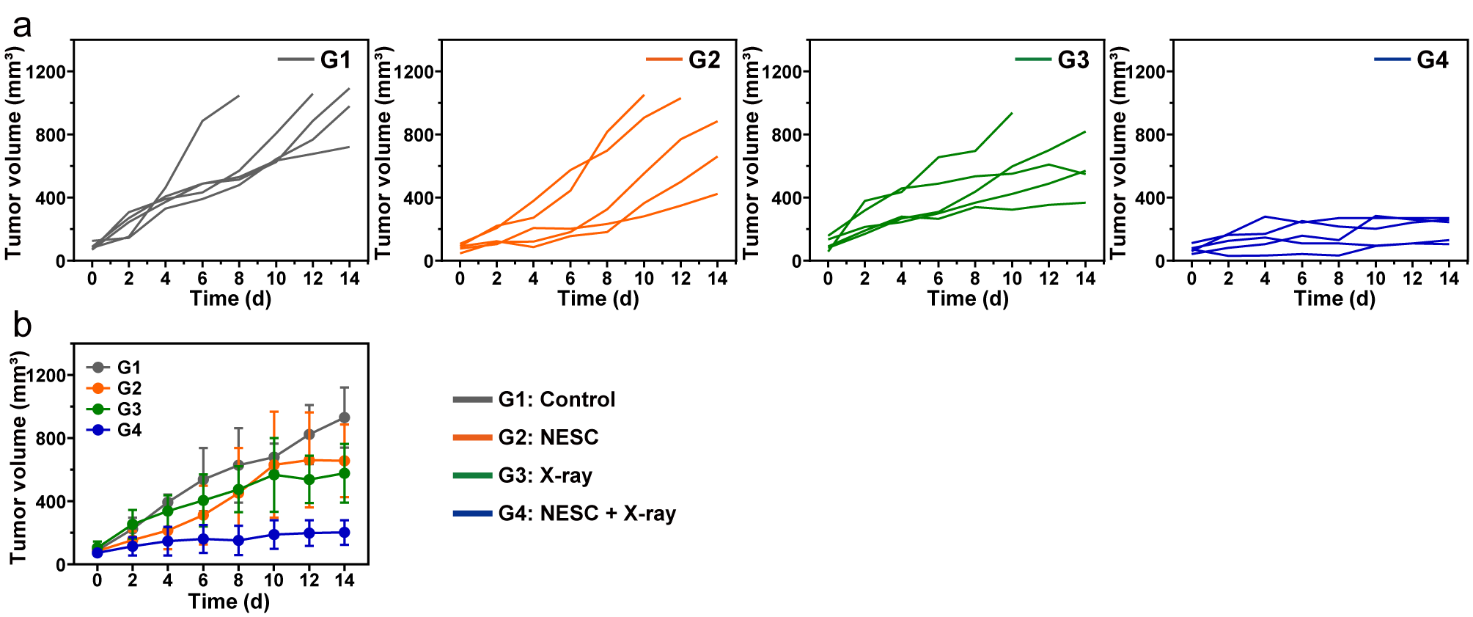
**

**Supplementary Fig. S8. Inhibitory effect of NESC on tumor progression in PDOX mice. a,b,** Respective tumor growth curves of each mouse (**a**) and each group (**b**) during 14 days.

**Supporting Tables**

**Supplementary Table 1 | Most upregulated genes identified in malignant cell population from GSE183904.**

| Names | Scores | Logfoldchanges | Pvals | Pvals_adj |
| --- | --- | --- | --- | --- |
| CLDN4 | 43.194897 | 4.1884284 | 0 | 0 |
| CLDN7 | 37.0827 | 4.7347436 | 5.3398E-301 | 2.5155E-297 |
| MDK | 35.852745 | 3.8396049 | 1.6666E-281 | 5.608E-278 |
| LGALS4 | 34.486977 | 2.1647415 | 1.2575E-260 | 3.7024E-257 |
| MISP | 33.78702 | 3.1479635 | 3.0589E-250 | 7.205E-247 |
| TPI1 | 33.76251 |  | 7.0063E-250 | 1.5002E-246 |
| HNRNPA2B1 | 33.29014 |  | 5.3631E-243 | 1.0527E-239 |
| CYC1 | 32.882988 | 3.9679685 | 3.848E-237 | 6.972E-234 |
| GPI | 32.65574 |  | 6.6429E-234 | 1.1176E-230 |
| ACTG1 | 32.421505 | 2.8824937 | 1.3662E-230 | 2.1452E-227 |
| KRT8 | 31.879343 | 1.3668933 | 5.1627E-223 | 7.6002E-220 |
| RAB11FIP1 | 31.737595 | 3.3517094 | 4.7096E-221 | 6.5252E-218 |
| TMPRSS4 | 31.445263 | 3.1723053 | 4.8727E-217 | 6.3763E-214 |
| ACTN4 | 31.301273 | 4.5166416 | 4.4837E-215 | 5.5584E-212 |
| DSP | 31.260082 | 2.3277667 | 1.6285E-214 | 1.9179E-211 |
| HNRNPU | 31.230148 |  | 4.1534E-214 | 4.6585E-211 |
| LMNA | 31.023912 |  | 2.5659E-211 | 2.7471E-208 |
| PKM | 30.992184 |  | 6.87E-211 | 7.0355E-208 |
| AMN | 30.878788 | 2.417861 | 2.3016E-209 | 2.1685E-206 |
| PRSS3 | 30.483442 | 2.7871351 | 4.3197E-204 | 3.7684E-201 |
| AGPAT2 | 30.211132 | 3.0292208 | 1.6916E-200 | 1.423E-197 |
| C15orf48 | 30.12357 | 2.4123902 | 2.381E-199 | 1.9339E-196 |
| USH1C | 30.062408 | 2.6730142 | 1.503E-198 | 1.18E-195 |
| JUP | 29.888144 | 2.1509829 | 2.806E-196 | 2.132E-193 |
| MYH14 | 29.733448 | 2.2508194 | 2.8388E-194 | 2.0895E-191 |
| H2AFY | 29.55433 |  | 5.7773E-192 | 4.1236E-189 |
| TNFRSF12A | 29.507812 |  | 2.2856E-191 | 1.5834E-188 |
| ETS2 | 29.483925 |  | 4.6276E-191 | 3.1143E-188 |
| MUC13 | 29.423883 | 3.385554 | 2.7182E-190 | 1.7784E-187 |
| CES2 | 29.332169 | 2.6776161 | 4.0344E-189 | 2.5683E-186 |
| SON | 29.325708 |  | 4.8772E-189 | 3.0231E-186 |
| BAIAP2L2 | 29.298595 | 3.4767797 | 1.0808E-188 | 6.5274E-186 |
| TRAF4 | 29.15356 | 2.9720929 | 7.5292E-187 | 4.4336E-184 |
| S100A6 | 29.129503 | 2.0543869 | 1.5191E-186 | 8.7272E-184 |
| HIST1H2AM | 29.114412 | 4.172813 | 2.3588E-186 | 1.3228E-183 |
| SFN | 29.072966 | 1.789694 | 7.8882E-186 | 4.3209E-183 |
| LRRFIP1 | 28.992281 |  | 8.2322E-185 | 4.4068E-182 |
| CSKMT | 28.950874 | 5.9924774 | 2.7361E-184 | 1.4321E-181 |
| HIST1H2AG | 28.94667 |  | 3.0906E-184 | 1.5825E-181 |
| PKP3 | 28.845745 | 2.021195 | 5.7293E-183 | 2.8275E-180 |

**Supplementary Table 2 | Clinical characteristics of enrolled GC patients.**

| **Groups** | **n (%)** |
| --- | --- |
| **Sex** |  |
| Male | 39 (69.6) |
| Female | 17 (30.4) |
| **Age (years)** |  |
| <60 | 5 (8.9) |
| ≥60 | 51 (91.1) |
| **Differentiation** |  |
| Moderate | 28 (50.0) |
| High | 28 (50.0) |
| **Tumor stage** |  |
| Stage Ⅰa | 4 (7.1) |
| Stage Ⅰb | 2 (3.6) |
| Stage Ⅱb | 9 (16.1) |
| Stage Ⅲa | 15 (26.8) |
| Stage Ⅲb | 12 (21.4) |
| Stage Ⅲc | 13 (23.2) |
| Stage Ⅳ | 1 (1.8) |
| **Total** | 56 (100) |

**Supplementary Table 3 | Proteins identified in NK-sEVs by liquid chromatography-mass spectrometry.**

| No. | Uniprot ID | No. | Uniprot ID | No. | Uniprot ID | No. | Uniprot ID | No. | Uniprot ID |
| --- | --- | --- | --- | --- | --- | --- | --- | --- | --- |
| 1 | P35579 | 51 | Q7Z6Z7 | 101 | P13796 | 151 | Q9BUJ2 | 201 | P43007 |
| 2 | Q00610 | 52 | P68366 | 102 | P12268 | 152 | P29692 | 202 | Q9UNF0 |
| 3 | P29144 | 53 | P78347 | 103 | O75717 | 153 | P56192 | 203 | Q7Z6I6 |
| 4 | Q9Y490 | 54 | Q9Y4L1 | 104 | P60228 | 154 | O75955 | 204 | Q29963 |
| 5 | P55072 | 55 | P10809 | 105 | P42768 | 155 | Q9Y3L3 | 205 | Q16666 |
| 6 | P49327 | 56 | P13612 | 106 | Q8N163 | 156 | P68400 | 206 | O94776 |
| 7 | Q14152 | 57 | P00558 | 107 | P23246 | 157 | P07996 | 207 | O95347 |
| 8 | P07814 | 58 | Q92835 | 108 | P00450 | 158 | P31939 | 208 | Q29RF7 |
| 9 | P21333 | 59 | P13639 | 109 | Q9UBW5 | 159 | Q12864 | 209 | Q10567 |
| 10 | P08238 | 60 | P17987 | 110 | P50991 | 160 | Q16181 | 210 | Q92598 |
| 11 | P08575 | 61 | P11388 | 111 | Q9UHD8 | 161 | Q8TAQ2 | 211 | P14317 |
| 12 | P14618 | 62 | P53396 | 112 | Q99832 | 162 | P13591 | 212 | P51531 |
| 13 | P41252 | 63 | P13645 | 113 | Q8TEQ6 | 163 | P01023 | 213 | P23229 |
| 14 | O75533 | 64 | Q13045 | 114 | Q9BZK7 | 164 | P11387 | 214 | P19823 |
| 15 | P08133 | 65 | P07355 | 115 | P35606 | 165 | Q13347 | 215 | Q92922 |
| 16 | P02751 | 66 | P02786 | 116 | Q13200 | 166 | Q15428 | 216 | Q00653 |
| 17 | P46940 | 67 | P13637 | 117 | P04075 | 167 | P50995 | 217 | P62879 |
| 18 | P07437 | 68 | Q71U36 | 118 | O15372 | 168 | P12259 | 218 | Q96CX2 |
| 19 | Q15393 | 69 | P49368 | 119 | Q8WWI5 | 169 | P12956 | 219 | P78562 |
| 20 | Q08211 | 70 | P22314 | 120 | P08670 | 170 | Q8NF50 | 220 | Q99873 |
| 21 | P55884 | 71 | P48643 | 121 | P26010 | 171 | P08195 | 221 | P13010 |
| 22 | P05023 | 72 | P14625 | 122 | P05106 | 172 | P63104 | 222 | P23396 |
| 23 | O43707 | 73 | P54136 | 123 | P00734 | 173 | Q92888 | 223 | O14744 |
| 24 | B0I1T2 | 74 | P12270 | 124 | Q12874 | 174 | Q9Y520 | 224 | P49591 |
| 25 | P50990 | 75 | P11940 | 125 | P27694 | 175 | O43242 | 225 | Q15019 |
| 26 | P07900 | 76 | Q13428 | 126 | P04899 | 176 | P15311 | 226 | P06396 |
| 27 | P68371 | 77 | P14868 | 127 | Q92945 | 177 | P35908 | 227 | O95490 |
| 28 | P04264 | 78 | P40227 | 128 | Q13435 | 178 | P68032 | 228 | P35637 |
| 29 | Q9Y262 | 79 | P36578 | 129 | P31146 | 179 | Q92841 | 229 | P06744 |
| 30 | P53621 | 80 | P68104 | 130 | P27487 | 180 | P39023 | 230 | P51532 |
| 31 | P60709 | 81 | Q8N8A2 | 131 | P26641 | 181 | P18124 | 231 | Q9UMS4 |
| 32 | P05107 | 82 | Q9P2J5 | 132 | P51610 | 182 | P62424 | 232 | Q01844 |
| 33 | Q9HC35 | 83 | Q04637 | 133 | P35527 | 183 | Q14103 | 233 | P47897 |
| 34 | P14866 | 84 | Q14697 | 134 | Q14141 | 184 | P30481 | 234 | P50395 |
| 35 | O75083 | 85 | Q13263 | 135 | P23634 | 185 | Q15021 | 235 | O60832 |
| 36 | P11142 | 86 | O43143 | 136 | Q9NTJ3 | 186 | P23526 | 236 | P62241 |
| 37 | Q99613 | 87 | Q9UI08 | 137 | Q09028 | 187 | Q15046 | 237 | P30492 |
| 38 | Q16555 | 88 | P04439 | 138 | Q16851 | 188 | Q8WWM7 | 238 | Q9H7D7 |
| 39 | P26038 | 89 | O95466 | 139 | Q8TD19 | 189 | P52272 | 239 | Q01518 |
| 40 | Q86XP3 | 90 | Q96BY6 | 140 | O43719 | 190 | O60271 | 240 | Q9H4M9 |
| 41 | P20701 | 91 | P51991 | 141 | P05362 | 191 | Q7L2H7 | 241 | Q14203 |
| 42 | Q32MZ4 | 92 | P13746 | 142 | P35611 | 192 | P0DMV9 | 242 | Q8IX12 |
| 43 | P11021 | 93 | P01008 | 143 | O00303 | 193 | Q00839 | 243 | P08754 |
| 44 | Q15459 | 94 | O15371 | 144 | P46013 | 194 | P62873 | 244 | P35613 |
| 45 | O60610 | 95 | P22234 | 145 | Q13283 | 195 | Q9UEY8 | 245 | P63244 |
| 46 | P43243 | 96 | P0C0L4 | 146 | Q15758 | 196 | Q8NI27 | 246 | P52907 |
| 47 | P34932 | 97 | P01024 | 147 | P01889 | 197 | P31943 | 247 | P07195 |
| 48 | P78371 | 98 | P27816 | 148 | Q15233 | 198 | O76094 | 248 | P08865 |
| 49 | P06733 | 99 | P50552 | 149 | O94804 | 199 | Q86UX7 | 249 | P27797 |
| 50 | P35580 | 100 | P04406 | 150 | Q92619 | 200 | Q7Z3B4 | 250 | O00267 |

| No. | Uniprot ID | No. | Uniprot ID | No. | Uniprot ID | No. | Uniprot ID | No. | Uniprot ID |
| --- | --- | --- | --- | --- | --- | --- | --- | --- | --- |
| 251 | P46777 | 301 | P48444 | 351 | P00736 | 401 | P61981 | 451 | Q14005 |
| 252 | P12109 | 302 | Q7Z406 | 352 | P62701 | 402 | P15880 | 452 | P60174 |
| 253 | O14776 | 303 | P35998 | 353 | P13747 | 403 | P61158 | 453 | Q9Y376 |
| 254 | Q9Y265 | 304 | O00232 | 354 | P53992 | 404 | P52209 | 454 | P62917 |
| 255 | O60506 | 305 | P09874 | 355 | P62805 | 405 | P49863 | 455 | P08236 |
| 256 | Q93099 | 306 | P61224 | 356 | P53618 | 406 | P49790 | 456 | Q14515 |
| 257 | P40222 | 307 | Q86X55 | 357 | Q8IUR7 | 407 | Q9C0C9 | 457 | O95104 |
| 258 | P06729 | 308 | Q9BPX3 | 358 | P27348 | 408 | Q9UPN7 | 458 | Q9Y2W1 |
| 259 | O94979 | 309 | Q16576 | 359 | Q02880 | 409 | P02774 | 459 | P30825 |
| 260 | Q14157 | 310 | Q08J23 | 360 | Q9Y678 | 410 | Q13155 | 460 | P09972 |
| 261 | Q92608 | 311 | O75821 | 361 | Q5T1M5 | 411 | Q15637 | 461 | P62979 |
| 262 | P02768 | 312 | Q9UJU6 | 362 | Q14974 | 412 | Q9UPN9 | 462 | P09651 |
| 263 | P62826 | 313 | P63010 | 363 | Q6Y7W6 | 413 | P05556 | 463 | Q99729 |
| 264 | P67809 | 314 | Q9BZQ8 | 364 | P28838 | 414 | Q8TDB8 | 464 | P26639 |
| 265 | Q9ULA0 | 315 | Q8WUM4 | 365 | Q9UQE7 | 415 | P30153 | 465 | P02679 |
| 266 | Q14444 | 316 | Q13409 | 366 | Q96AE4 | 416 | O15117 | 466 | Q9Y5A9 |
| 267 | Q9UJX2 | 317 | P17844 | 367 | P69905 | 417 | P57678 | 467 | Q9Y6R7 |
| 268 | P28907 | 318 | Q96SB4 | 368 | Q14839 | 418 | O43390 | 468 | Q9UIQ6 |
| 269 | P11586 | 319 | Q6NYC8 | 369 | Q15424 | 419 | Q9NQC3 | 469 | P49736 |
| 270 | P30101 | 320 | O60814 | 370 | Q15020 | 420 | Q8IWX8 | 470 | P16070 |
| 271 | Q99829 | 321 | Q9NVA2 | 371 | P30876 | 421 | Q8NG11 | 471 | Q9H2H9 |
| 272 | Q14694 | 322 | O14974 | 372 | Q92973 | 422 | P17812 | 472 | Q969P0 |
| 273 | Q9UN86 | 323 | Q9P2E9 | 373 | P11171 | 423 | Q13547 | 473 | P11166 |
| 274 | Q9Y230 | 324 | Q8N743 | 374 | P06899 | 424 | Q92900 | 474 | P61026 |
| 275 | Q9H4B7 | 325 | Q9UHY1 | 375 | P61313 | 425 | P62333 | 475 | Q14117 |
| 276 | Q9NPH2 | 326 | Q9P265 | 376 | P62195 | 426 | Q9BVL2 | 476 | Q9NUV9 |
| 277 | P61247 | 327 | P09326 | 377 | Q92925 | 427 | Q5H9R7 | 477 | P53985 |
| 278 | P23528 | 328 | P23588 | 378 | P20810 | 428 | P60842 | 478 | P07948 |
| 279 | P00338 | 329 | O14980 | 379 | Q9UNM6 | 429 | P61978 | 479 | Q8WUX1 |
| 280 | Q14980 | 330 | Q00577 | 380 | Q02878 | 430 | Q8NE71 | 480 | Q6PKG0 |
| 281 | Q9UHB9 | 331 | Q96CN7 | 381 | Q02543 | 431 | Q9NR30 | 481 | Q15942 |
| 282 | Q9UHN6 | 332 | P07358 | 382 | P07910 | 432 | P54709 | 482 | P07237 |
| 283 | P37198 | 333 | O75366 | 383 | Q9Y6Y8 | 433 | P13671 | 483 | Q9Y3I0 |
| 284 | O60841 | 334 | Q15008 | 384 | Q12996 | 434 | P98175 | 484 | Q9UJX3 |
| 285 | P12110 | 335 | P78527 | 385 | Q14683 | 435 | Q14978 | 485 | Q13247 |
| 286 | P27105 | 336 | Q13148 | 386 | P05546 | 436 | Q9Y240 | 486 | Q07955 |
| 287 | P11413 | 337 | P08758 | 387 | Q16531 | 437 | P04004 | 487 | P50914 |
| 288 | Q9NZN3 | 338 | Q15029 | 388 | Q86U42 | 438 | Q8TDB6 | 488 | P09619 |
| 289 | P17655 | 339 | P19338 | 389 | P33176 | 439 | O00231 | 489 | P62136 |
| 290 | E9PAV3 | 340 | P27824 | 390 | P51665 | 440 | Q5JWF2 | 490 | Q16819 |
| 291 | Q14520 | 341 | P31946 | 391 | P18621 | 441 | P49257 | 491 | P55058 |
| 292 | P62258 | 342 | P26640 | 392 | P04908 | 442 | Q5T6F2 | 492 | O15162 |
| 293 | P62834 | 343 | Q9NZT2 | 393 | Q9P258 | 443 | P00738 | 493 | P49747 |
| 294 | P22102 | 344 | Q14254 | 394 | P00488 | 444 | Q9H223 | 494 | Q9BQA1 |
| 295 | P46379 | 345 | P49756 | 395 | P13807 | 445 | O43516 | 495 | P43686 |
| 296 | Q9Y6G9 | 346 | P15170 | 396 | Q8WXF1 | 446 | P02765 | 496 | P07384 |
| 297 | P08567 | 347 | P02675 | 397 | Q99460 | 447 | Q13576 | 497 | P22352 |
| 298 | P54577 | 348 | Q92878 | 398 | P01860 | 448 | Q13094 | 498 | Q12768 |
| 299 | Q8NC51 | 349 | P01031 | 399 | P62191 | 449 | P18206 | 499 | Q96S59 |
| 300 | P20742 | 350 | Q07020 | 400 | O00299 | 450 | P27708 | 500 | P02748 |

| No. | Uniprot ID | No. | Uniprot ID | No. | Uniprot ID | No. | Uniprot ID | No. | Uniprot ID |
| --- | --- | --- | --- | --- | --- | --- | --- | --- | --- |
| 501 | Q92499 | 551 | Q12904 | 601 | Q96RS6 | 651 | O95232 | 701 | P01591 |
| 502 | P02794 | 552 | P15153 | 602 | Q96B97 | 652 | P02042 | 702 | Q15554 |
| 503 | P55209 | 553 | P19838 | 603 | P49588 | 653 | O75695 | 703 | Q16563 |
| 504 | P47710 | 554 | P36871 | 604 | P61073 | 654 | O15260 | 704 | Q9BXJ9 |
| 505 | P84103 | 555 | O60488 | 605 | Q9Y5K6 | 655 | Q13469 | 705 | Q9GZT8 |
| 506 | Q8WW12 | 556 | P02656 | 606 | P09543 | 656 | Q12907 | 706 | A0A0B4J2H0 |
| 507 | O14672 | 557 | Q96ST3 | 607 | P46776 | 657 | P51149 | 707 | Q15848 |
| 508 | P10643 | 558 | P30260 | 608 | O75400 | 658 | Q92896 | 708 | P04632 |
| 509 | P62820 | 559 | Q14566 | 609 | Q15907 | 659 | Q13201 | 709 | Q3YEC7 |
| 510 | Q2M389 | 560 | Q9UKE5 | 610 | Q16630 | 660 | Q9H307 | 710 | P40429 |
| 511 | Q15833 | 561 | Q86UE4 | 611 | Q9UPT8 | 661 | P61289 | 711 | Q86VM9 |
| 512 | Q9UQ80 | 562 | O75530 | 612 | P20839 | 662 | Q92905 | 712 | O43290 |
| 513 | O96019 | 563 | Q12805 | 613 | Q16637 | 663 | Q6IN85 | 713 | P49959 |
| 514 | P26373 | 564 | Q9UMX0 | 614 | P47756 | 664 | Q05682 | 714 | Q96KP4 |
| 515 | P55060 | 565 | Q13042 | 615 | P60953 | 665 | Q66K74 | 715 | P41440 |
| 516 | O00506 | 566 | Q9UGI8 | 616 | P50453 | 666 | Q9H1A4 | 716 | P09871 |
| 517 | Q92734 | 567 | P15529 | 617 | Q05048 | 667 | P05155 | 717 | Q6DN90 |
| 518 | Q14344 | 568 | P46781 | 618 | O76074 | 668 | Q15003 | 718 | Q5VW36 |
| 519 | O15042 | 569 | Q8IYB5 | 619 | P26358 | 669 | P38919 | 719 | Q9Y295 |
| 520 | O43776 | 570 | P61106 | 620 | O43760 | 670 | P62249 | 720 | Q8WVC0 |
| 521 | P29401 | 571 | Q9Y3P9 | 621 | P02647 | 671 | O95782 | 721 | Q15007 |
| 522 | O75131 | 572 | P50454 | 622 | P07947 | 672 | Q9ULV4 | 722 | Q7Z2W4 |
| 523 | Q9UPU7 | 573 | Q9P1W8 | 623 | Q8NBJ4 | 673 | Q15582 | 723 | P04040 |
| 524 | O75175 | 574 | P08134 | 624 | Q00341 | 674 | Q96F15 | 724 | P17252 |
| 525 | Q13838 | 575 | P47755 | 625 | O60711 | 675 | Q96QR8 | 725 | Q8TB24 |
| 526 | Q12906 | 576 | Q15427 | 626 | Q9UKY7 | 676 | Q06210 | 726 | P02749 |
| 527 | Q96F07 | 577 | Q99542 | 627 | A0A087WW87 | 677 | P08697 | 727 | Q13418 |
| 528 | Q7KZF4 | 578 | P15927 | 628 | Q14204 | 678 | Q12905 | 728 | P62280 |
| 529 | P00747 | 579 | Q9BY44 | 629 | O00187 | 679 | Q86YP4 | 729 | Q13011 |
| 530 | P33241 | 580 | P62937 | 630 | P48634 | 680 | Q01433 | 730 | P20700 |
| 531 | P26368 | 581 | Q13098 | 631 | P19623 | 681 | P17980 | 731 | O00186 |
| 532 | Q8IWA5 | 582 | P57737 | 632 | P08514 | 682 | P62753 | 732 | O75915 |
| 533 | Q63HN8 | 583 | P48059 | 633 | P05388 | 683 | P61201 | 733 | Q96HN2 |
| 534 | O00743 | 584 | P13798 | 634 | P02671 | 684 | Q14677 | 734 | Q9HC16 |
| 535 | Q15043 | 585 | Q15018 | 635 | Q99805 | 685 | Q8WUM9 | 735 | Q14671 |
| 536 | O75822 | 586 | P51674 | 636 | P50851 | 686 | P42566 | 736 | Q9Y266 |
| 537 | Q99567 | 587 | Q96D96 | 637 | P05198 | 687 | Q9NX58 | 737 | P30740 |
| 538 | P02788 | 588 | P46778 | 638 | Q2TAY7 | 688 | P50502 | 738 | P01619 |
| 539 | P31948 | 589 | Q99808 | 639 | Q16543 | 689 | A0A0C4DH25 | 739 | O14497 |
| 540 | P04424 | 590 | P30566 | 640 | Q07666 | 690 | P01009 | 740 | P15121 |
| 541 | P18669 | 591 | Q9BXS5 | 641 | Q08945 | 691 | Q99570 | 741 | P55786 |
| 542 | P20073 | 592 | P06737 | 642 | P19827 | 692 | P68431 | 742 | O15127 |
| 543 | P23381 | 593 | P46782 | 643 | O95197 | 693 | Q9NTZ6 | 743 | Q9P2N5 |
| 544 | Q9Y5B9 | 594 | P11216 | 644 | P33991 | 694 | P12111 | 744 | P41091 |
| 545 | Q7L014 | 595 | P0DOX5 | 645 | P06748 | 695 | Q13867 | 745 | P12429 |
| 546 | P13646 | 596 | P20702 | 646 | P09525 | 696 | P61221 | 746 | P62269 |
| 547 | Q0VD83 | 597 | P13640 | 647 | P33240 | 697 | O75874 | 747 | O00160 |
| 548 | O43747 | 598 | Q14699 | 648 | Q8WU90 | 698 | O95373 | 748 | P48426 |
| 549 | Q96PK6 | 599 | P48740 | 649 | P52566 | 699 | P09211 | 749 | Q92797 |
| 550 | Q969G3 | 600 | Q14624 | 650 | P61353 | 700 | Q9H773 | 750 | O75153 |

| No. | Uniprot ID | No. | Uniprot ID | No. | Uniprot ID | No. | Uniprot ID | No. | Uniprot ID |
| --- | --- | --- | --- | --- | --- | --- | --- | --- | --- |
| 751 | Q2M2I8 | 801 | P49908 | 851 | P32969 | 901 | Q9NQ25 | 951 | Q9H2S9 |
| 752 | P02649 | 802 | Q9Y3Q8 | 852 | Q5T4S7 | 902 | P53801 | 952 | O75475 |
| 753 | Q9C0J8 | 803 | O75891 | 853 | P05156 | 903 | Q8NBI5 | 953 | P30419 |
| 754 | P18428 | 804 | Q9UQP3 | 854 | Q13033 | 904 | Q9Y6F7 | 954 | Q9H2U1 |
| 755 | P04114 | 805 | Q15061 | 855 | P06727 | 905 | Q9H2U2 | 955 | Q8WZ42 |
| 756 | Q9NUQ3 | 806 | P55010 | 856 | P36888 | 906 | P61204 | 956 | Q92620 |
| 757 | Q08722 | 807 | P02746 | 857 | P84098 | 907 | Q6N022 | 957 | Q9BYW2 |
| 758 | P26599 | 808 | Q9NSD9 | 858 | Q9BS26 | 908 | Q14C86 | 958 | Q08380 |
| 759 | P46063 | 809 | Q9BUL8 | 859 | Q8NF91 | 909 | A0A0B4J1V0 | 959 | O14641 |
| 760 | Q9Y6C2 | 810 | P31350 | 860 | Q9UJX5 | 910 | Q13303 | 960 | P49763 |
| 761 | Q14761 | 811 | O14828 | 861 | Q9Y487 | 911 | Q08378 | 961 | P00966 |
| 762 | O00155 | 812 | P51114 | 862 | P01701 | 912 | A0A075B6J6 | 962 | Q9Y4F3 |
| 763 | P52732 | 813 | Q86VP6 | 863 | Q52LJ0 | 913 | O43826 | 963 | A5YKK6 |
| 764 | Q7Z4I7 | 814 | Q01650 | 864 | Q06830 | 914 | P07357 |  |  |
| 765 | Q99572 | 815 | P07360 | 865 | P04070 | 915 | Q9P2I0 |  |  |
| 766 | Q9UKM9 | 816 | O14745 | 866 | Q15022 | 916 | Q9NVX2 |  |  |
| 767 | Q96I25 | 817 | P16671 | 867 | P55735 | 917 | Q7KZ85 |  |  |
| 768 | Q5BKZ1 | 818 | Q7L5N1 | 868 | Q9BSQ5 | 918 | P23142 |  |  |
| 769 | A6NC98 | 819 | P22059 | 869 | Q15910 | 919 | Q9UHG3 |  |  |
| 770 | O75636 | 820 | Q68EM7 | 870 | P61254 | 920 | Q13023 |  |  |
| 771 | Q14847 | 821 | O00154 | 871 | Q14676 | 921 | O43670 |  |  |
| 772 | Q9BZE4 | 822 | Q13642 | 872 | Q96IY4 | 922 | Q8N9N7 |  |  |
| 773 | Q15404 | 823 | Q7Z460 | 873 | Q9BQS7 | 923 | Q9ULT0 |  |  |
| 774 | Q9H2K8 | 824 | Q8IUI8 | 874 | H7BZ55 | 924 | P62910 |  |  |
| 775 | Q8ND56 | 825 | P55083 | 875 | P52594 | 925 | P14210 |  |  |
| 776 | Q9UHI6 | 826 | Q8N1G4 | 876 | Q8NDX1 | 926 | Q15437 |  |  |
| 777 | Q9UKX7 | 827 | Q9BX63 | 877 | O75534 | 927 | Q96JM3 |  |  |
| 778 | P57721 | 828 | Q9UBQ5 | 878 | Q16186 | 928 | A0A075B6H9 |  |  |
| 779 | O00487 | 829 | P22626 | 879 | Q8TC71 | 929 | Q15024 |  |  |
| 780 | A0A075B6K0 | 830 | Q15477 | 880 | O15144 | 930 | A0A0B4J1U7 |  |  |
| 781 | O15294 | 831 | Q8N684 | 881 | O75643 | 931 | P00742 |  |  |
| 782 | Q04206 | 832 | Q93088 | 882 | P27169 | 932 | Q96EP5 |  |  |
| 783 | P10909 | 833 | Q16610 | 883 | P19397 | 933 | Q9Y5Y0 |  |  |
| 784 | P78344 | 834 | Q9NWH9 | 884 | Q567U6 | 934 | Q13123 |  |  |
| 785 | P16150 | 835 | A0A0C4DH55 | 885 | Q8IZL8 | 935 | Q14517 |  |  |
| 786 | P55036 | 836 | P02730 | 886 | P40818 | 936 | O14802 |  |  |
| 787 | P62906 | 837 | Q8IXQ6 | 887 | P49840 | 937 | P04217 |  |  |
| 788 | Q99661 | 838 | P46779 | 888 | P04003 | 938 | Q14511 |  |  |
| 789 | Q96GM5 | 839 | Q8IWT3 | 889 | P33527 | 939 | P83881 |  |  |
| 790 | P48960 | 840 | P60033 | 890 | Q9UK55 | 940 | P00740 |  |  |
| 791 | O95602 | 841 | P00390 | 891 | Q8NHY5 | 941 | P55160 |  |  |
| 792 | Q9H2P0 | 842 | P0DOX8 | 892 | Q13144 | 942 | P15104 |  |  |
| 793 | P00352 | 843 | Q9Y4E8 | 893 | O43399 | 943 | P23467 |  |  |
| 794 | P08621 | 844 | Q9BXP5 | 894 | P04843 | 944 | Q9NZM3 |  |  |
| 795 | Q96S55 | 845 | P49915 | 895 | Q86UP2 | 945 | Q9H6Z4 |  |  |
| 796 | P31323 | 846 | Q9P2B2 | 896 | Q5R372 | 946 | Q9Y2G4 |  |  |
| 797 | O94906 | 847 | P49815 | 897 | Q8N475 | 947 | P51805 |  |  |
| 798 | P81605 | 848 | Q9P1T7 | 898 | Q8IU68 | 948 | P39748 |  |  |
| 799 | Q9BXB4 | 849 | Q6P2Q9 | 899 | Q14161 | 949 | Q7Z5K2 |  |  |
| 800 | P48052 | 850 | Q01538 | 900 | Q02790 | 950 | P46976 |  |  |
